# Interventions for Improving Informal Social Support for Victim‐Survivors of Domestic Violence and Abuse: An Evidence and Gap Map

**DOI:** 10.1002/cl2.70026

**Published:** 2025-04-16

**Authors:** Karen L. Schucan Bird, Nicola Stokes, Carol Rivas

**Affiliations:** ^1^ Social Research Institute, University College London London UK; ^2^ Formerly SafeLives Bristol UK

## Abstract

**Background:**

Domestic Violence and Abuse (DVA) is a significant global problem that warrants a robust, multi‐sectoral response. The Covid‐19 pandemic highlighted that informal and social networks play a critical role in responding to victim‐survivors, alongside formal agencies and specialist services. Friends, relatives, neighbours and colleagues are uniquely placed to recognise abuse, respond and refer to wider services, where appropriate. Seeking to harness this potential, interventions tailored towards such informal supporters are being developed and implemented around the world. Yet little is known about such interventions. By pulling together the research on such programmes, this evidence and gap map begins to advance the understanding of informal support interventions, pinpointing the range and type of interventions implemented around the world, and the extent of the available evidence. This provides valuable insights for policy makers and practitioners seeking to commission or develop interventions and research in this area, with a view to facilitating a holistic, societal‐wide response to domestic abuse. The evidence and gap map was a collaboration of academics and specialists, as well as domestic abuse researchers, with input and guidance from an Advisory Group.

**Objectives:**

This evidence and gap map aims to establish the nature and extent of the empirical primary research on interventions aiming to create or enhance informal support for victim‐survivors of domestic abuse, identifying clusters of evidence potentially suitable for synthesis, and gaps in the research base.

**Search Methods:**

The following bibliographic databases were searched for published studies from inception to 31st October 2022: APA PsycINFO, Social Policy and Practice, ASSIA, PubMed, and Social Science Citation Index. Identifying grey literature was an important pillar of the search strategy and so websites of domestic abuse organisations, predominantly in the United Kingdom, were also searched. Similarly, a targeted search of specialist systematic review, policy and domestic abuse databases was undertaken from inception to 10th July 2023.

**Selection Criteria:**

The evidence and gap map focused on any interventions that explicitly aimed to create or enhance informal social support for victim‐survivors of domestic abuse. Eligible interventions targeted the providers of the support (i.e., friends, relatives, neighbours or colleagues), the victim‐survivor, the relationship between them, and/or the wider community within which the informal support was provided. All study designs were included, reporting qualitative or quantitative data for samples or victim‐survivors (adults who were/had been experiencing abuse in an intimate relationship) or informal supporters. Outcomes were not used as part of the eligibility criteria. Eligible studies needed to be published in English.

**Data Collection and Analysis:**

All studies included in the evidence and gap map were coded by two independent reviewers, using specialist systematic review software EPPI Reviewer. Details were collected about the study sample, study design, intervention and outcomes. Quality appraisal was not undertaken.

**Main Results:**

The EGM identified 47 primary studies of interventions that aimed to create, enhance or facilitate informal support for victim‐survivors of domestic violence and abuse. The overwhelming majority of evidence is drawn from the Global North, and there is dissonance between the small evidence base and the relatively larger number of informal support interventions implemented around the world. The EGM highlights the importance of diverse study designs and grey literature in this field. The body of research is unevenly distributed, with the greatest concentration of studies around interventions directed towards victim‐survivors, such as support groups or mentoring, and those tailored towards informal supporters, such as education and training. Most research reported on female, adult victim‐survivors with a particular emphasis on their mental health and wellbeing, and their help‐seeking behaviours. The reporting of such outcomes aligns with wider service user/provider priorities and highlights the imperative of DVA research to improve the lives of victim‐survivors. The EGM found little research focused on interventions targeting structural factors that shape informal support, such as social relationships or community norms, and a lack of data on specific population groups including victim‐survivors in the longer term, ethnic minority groups and men. There are major gaps in the research for informal supporters with limited data or outcomes for this group, and specific types of informal supporters (namely friends and family members) are notably absent from samples. The EGM also highlights a gap in the research on community‐level outcomes.

**Authors' Conclusions:**

To our knowledge, this EGM is the first to provide a comprehensive and rigorous overview of the evidence on informal support interventions in domestic abuse. The EGM provides a valuable tool for policymakers, practitioners and researchers seeking to navigate the evidence around such interventions. Whilst the EGM provides a partial picture of interventions around the world, the studies offer insight into informal support for victim‐survivors of DVA and the potential effects of intervening. The suite of interventions covered by the EGM can inspire policymakers to broaden the response to domestic abuse beyond frontline services, identify stakeholders and commission pilot studies to further understanding of informal support interventions. The evidence base can be strengthened with additional studies examining interventions that target relationships and communities, as well as individuals, and assessing a wider range of population groups. At the same time, the EGM offers pockets of rich data, such as outcomes on victim‐survivor mental health or interventions in faith‐based organisations, which can be utilised to inform current and future service provision.

**Abbreviations**
DVA: domestic violence and abuse
EGM: evidence and gap map
ISS: informal social support
NGO: non‐governmental organisation

## 1 Plain Language Summary

Evidence on interventions that create or enhance informal support for victim‐survivors of domestic violence and abuse (DVA) is concentrated in the Global North, and unevenly distributed across types of intervention, population and outcome.

### 1.1 The Evidence and Gap Map (EGM) in Brief

The relatively small evidence base includes diverse interventions from around the world, but research is concentrated on interventions tailored towards victim‐survivors' ability to create or foster informal support, primarily in the Global North. Studies focus on female, adult, victim‐survivors with a particular emphasis on their mental health and wellbeing, and help‐seeking.

### 1.2 What Is This EGM About?

People who experience violent and abusive relationships often rely on their friends, relatives, neighbours or work colleagues for help and support. Recognised as ‘informal supporters’, such groups play a critical role, but they often do not know what to do, feel helpless or fear getting involved. Seeking to address some of these barriers, non‐governmental organisations, communities and workplaces are beginning to develop programmes and activities to foster or strengthen support from friends, neighbours, colleagues and community leaders.

This EGM identifies studies about such interventions to better understand these programmes and build a picture of the research knowledge we have about them. This EGM helps decision‐makers to understand the extent and type of informal support interventions delivered around the world, enable them to assess existing approaches and so inform commissioning of further activities.

### 1.3 What Is The Aim of This EGM?

This EGM identifies and describes all the available evidence on interventions that aim to create or enhance informal support for people experiencing abusive relationships. In doing so, this EGM aims to provide data that can contribute to the development of policy and interventions that strengthen societal responses to violence and abuse.

### 1.4 What Studies Are Included?

The EGM includes any research providing qualitative and/or quantitative data on informal social support (ISS) interventions, published in English. Eligible studies need to report data for informal supporters or victim‐survivors of DVA (adults who were/had been experiencing abuse in an intimate relationship).

Studies are categorised according to whether the intervention was targeted towards informal supporters, the victim‐survivor, the relationship between them and/or the wider community. Any type of intervention is included so long as it has an explicit aim to enhance or promote informal support. Any form of informal support is eligible so long as it is provided by friends, colleagues, neighbours or community members, current non‐abusive partners or any family member. Studies can come from any region of the world and report any outcome.

The EGM includes 47 studies: 17 qualitative studies, 12 mixed method approaches (integrating qualitative and quantitative methods) 11 randomised controlled trials, 7 nonrandomised experimental designs and 5 descriptive quantitative studies.

### 1.5 What Are the Main Findings of This Gap Map?

The evidence base is small, relative to the larger number of informal support interventions being delivered around the world, and most of the research comes from North America, with a handful of studies also conducted in Europe, Asia and Africa.

The studies are unevenly distributed across intervention types, with clusters of evidence concentrated around interventions directed towards victim‐survivors of abuse, such as support groups or mentoring, and interventions targeting informal supporters, such as education and training. The EGM found little research focused on interventions directed towards social relationships or communities.

Most studies report data on adult female victim‐survivors, with a particular emphasis on their mental health and wellbeing, and help‐seeking behaviours. Such outcomes align with wider service user/provider priorities and highlight the imperative of DVA research to improve the lives of victim‐survivors. However, there are gaps in the data about specific groups of victim‐survivors, including those who have left the abusive relationship in the longer term, ethnic minorities and men. There is also limited data on informal supporters per se and particularly for friends and family members. The EGM also highlights a gap in research on community‐level outcomes.

### 1.6 What Do the Findings of the Map Mean?

To our knowledge, this EGM is the first to provide a comprehensive and rigorous overview of the current research on interventions that seek to create or enhance informal support for victim‐survivors of domestic abuse. This presents an initial step towards understanding these interventions, assessing their viability and effectiveness. The EGM therefore provides information for policymakers and practitioners seeking to prioritise and enhance informal support as part of a societal‐wide response to domestic abuse.

The evidence base can be strengthened with additional studies examining interventions that focus on relationships and communities and assessing a wider range of population groups.

### 1.7 How Up‐to‐Date Is This EGM?

The EGM includes academic research that was published up until the end of October 2022, and wider reports up until July 2023.

## 2 Background

### 2.1 The Problem

Domestic Violence and Abuse (DVA) is a serious, global problem. Estimates from the World Health Organisation suggest that a third of women around the world have experienced physical or sexual violence whilst in an intimate relationship (WHO 2021). Violence and abuse within relationships leads to serious social, health and economic consequences for individuals and society (Ellsberg et al. 2008; Oliver et al. 2019) and warrants a strong, multi‐sectoral response.

The Covid‐19 pandemic provided further impetus to take action against DVA, defined as psychological, physical, sexual, financial, controlling, coercive and/or threatening abuse from a current or former intimate partner (Domestic Abuse Act 2021). Data from around the world highlighted the intensification of DVA during the pandemic, especially against women and girls (UN Women 2020). Covid mitigation policies increased women's vulnerability to DVA through stay‐at‐home mandates alongside wider barriers that impeded access to their usual community resources and healthcare services (Nordhues et al. 2021). At the same time, policing, health and frontline DVA agencies faced a number of challenges in identifying and supporting individuals who were experiencing relationship abuse. Anecdotal evidence suggested that victim‐survivors were less able and/or willing to seek help from sources of formal support (services provided by the state, non‐governmental organisations and the legal system) (Peterman et al. 2020) and specialist DVA services faced increased demands. Within the United Kingdom, Refuge (domestic abuse organisation) saw a 700% increase in the number of visits to their Helpline website during the first ‘lockdown’ in March 2020 (Office for National Statistics 2020). In this context, victim‐survivors of DVA identified the importance of informal sources of support (friends, family, colleagues or community members) during the pandemic. Whilst victim‐survivors reported that they felt increasingly isolated from key sources of social support (Brodie et al. 2022), informal supporters also found creative ways to maintain contact and offer support during the pandemic (Gregory and Williamson 2021).

The role of informal supporters during the pandemic is indicative of the wider, crucial role that informal and social networks play in responding to victim‐survivors of abuse. For those ‘living with domestic abuse… the view from outside, from supportive friends, family and neighbours, is so important’ (DVA Survivor, SafeLives unpublished data). The majority of victim‐survivors eventually disclose to at least one informal supporter (Johnson and Belenko 2019; Sylaska and Edwards 2014), typically following a long period of abuse (SafeLives 2020). The Crime Survey for England and Wales in 2023, for example, reported that 70% of victims told someone they knew personally (e.g., friend or relative) about the abuse (ONS 2023).

Research suggests that female, young and marginalised groups are most likely to rely on, and disclose to, informal sources of support. Women are more likely to disclose to informal supporters than men (Sylaska and Edwards 2014) and younger victim‐survivors express a clear preference for informal rather than formal sources of support (Bundock et al. 2018; Moore et al. 2015; Sylaska and Edwards 2014). Ethnic and racial minorities are more likely to use informal rather than formal channels of support, pointing to the importance of immediate family and friends for help‐seeking (Fiolet et al. 2019; Ragavan et al. 2018; Rizo et al. 2011). In the United Kingdom, Black, Asian and racially minoritised women highlight the importance of informal social networks when mainstream services (e.g., police) are unsupportive (Femi‐Ajao et al. 2018). Socio‐economic status (SES) also influences the use of informal social support with individuals from middle/higher SES more likely to disclose to family or friends than their lower SES counterparts (Sylaska and Edwards 2014). Informal supporters therefore play a critical function in providing support to victim‐survivors, especially for particular groups.

Studies highlight that helpful informal support is associated with a range of improved outcomes for victim‐survivors. Evidence suggests that the provision of emotional and tangible support can empower victim‐survivors, and positive responses from family and friends can lead to improvements in their mental and physical health (Coker et al. 2002; Goodman and Smyth 2011; Sylaska and Edwards 2014; Weeks and LeBlanc 2011). Indeed, informal support and social networks are widely recognised as playing a key role in enabling the disclosure of abuse and facilitating help‐seeking from formal agencies (Kim and Royle 2023; Morgan et al. 2016; Sylaska and Edwards 2014). More widely, informal support constitutes an important pillar in societal‐wide responses to DVA. Governments in the United Kingdom and internationally acknowledge the importance of informal social support for victim‐survivors of DVA (e.g., HM Government Home Office 2019; Australian Institute Of Health And Welfare 2019; Government of Canada Department of Justice 2006) although policy initiatives tend to focus primarily on the delivery of support through formal channels (such as criminal justice responses). Within the health and domestic abuse sectors, there is growing recognition that practitioners can work to activate and mobilise social support to promote positive and sustained change (Goodman et al. 2015; WHO 2013).

Yet, whilst informal supporters play a critical role in responding to DVA, several factors shape the provision of support and the nature of the response to disclosures of abuse. There are multiple barriers to providing informal support (Latta and Goodman 2011). Studies highlight that informal supporters may feel helpless (Goodkind et al. 2003), fear retaliation from the perpetrator (Melgar et al. 2021) or do not know the best way to help (Gregory et al. 2017; Latta and Goodman 2011; McKenzie et al. 2020). Further, victim‐survivors report that informal responses are not always helpful (Nolet et al. 2020; Sylaska and Edwards 2014) and such responses are associated with poorer health and wellbeing of the victim (Dworkin et al. 2019). Therefore, there is scope for intervention in informal and social networks to maximise positive responses to disclosures of abuse and address barriers to the provision of helpful support.

### 2.2 The Intervention

Informal social support (ISS) interventions explicitly target the provision of support to individuals who are/have been experiencing violence and abuse in an intimate relationship. These interventions include ‘systematic activities designed to change the existing quality, level or function of an individual's personal social network or to create new networks and relationships’ (Budde and Schene 2004, 342). Interventions in DVA are commonly understood to operate on three different levels: primary (preventing the initiation or onset of abuse), secondary (identifying and responding to victim‐survivors) and tertiary (responding to victim‐survivors in the longer term and supporting recovery) (Trabold et al. 2018). ISS interventions are principally understood as secondary and tertiary forms of intervention, that is, systematic activities that seek to enhance the informal response to victim‐survivors of DVA (rather than activities that aim to prevent the initiation of abuse).

Informal social networks can be classified into different levels depending on the strength and proximity of the relationship with the victim‐survivor: primary (immediate friends, family, community and work colleagues), intermediate (peers and acquaintances) and tertiary levels (individuals within formal organisations and institutions) (Arón and Lorion 2003; França et al. 2018). Informal social support interventions are principally focused on the inter‐personal relationships that operate in the primary and intermediate social networks. These interventions explicitly aim to create or enhance ISS, which can take many forms, including emotional, informational and/or practical. Interventions create or enhance ISS by targeting the providers of the ISS (such as training to improve colleagues' awareness of, and responses to, domestic abuse within the workplace), the victim‐survivor (such as programmes that help victim‐survivors to reconnect with social networks) or the relationship between them (such as guidance for family and friends on how to respond to disclosures of abuse). Interventions can also aim to change the wider community within which ISS is provided (such as educational campaigns targeted at the community at large to challenge myths or raise awareness about DVA to mobilise support for individuals experiencing abuse).

ISS interventions are understood to work in several ways. Interventions can alter the knowledge, attitudes and behaviour of friends, family and wider support networks to improve their awareness and response to domestic abuse. ISS interventions can also directly bolster social support for victim‐survivors by leading to changes in the structure, function or dynamics of their social network (França et al. 2018). Strengthened support is associated with improved access to resources and help‐seeking behaviours of victim‐survivors (Kennedy et al. 2012; Liang et al. 2005; Zapor et al. 2015). Helpful responses to disclosures of abuse are also linked to several positive outcomes for the victim‐survivor, including improvements in quality of life and mental health (Beeble et al. 2009; Dworkin et al. 2019; Levendosky et al. 2004; Sylaska and Edwards 2014). However, ISS interventions can also potentially lead to adverse outcomes if the support is perceived to be unhelpful at the time, and/or changes in informal support lead to negative impacts (e.g., repercussions from the perpetrator). Nevertheless, there is growing recognition that informal support has an important role to play alongside formal services and agencies in a complex system of responses (Goodman et al. 2015; Sullivan 2017).

### 2.3 Why It Is Important to Develop the Evidence and Gap Map (EGM)

ISS interventions are both numerous and diverse. Around the world, there are multiple examples of informal support interventions in DVA. Examples include a global movement #ImamsForShe (that highlights the responsibility of Muslim leaders to address violence against women), Employers Initiative on Domestic Abuse in the United Kingdom and community mobilisation activities in India (Society for Nutrition, Education and Health Action in Mumbai). Yet, there is limited knowledge about the extent of the research on such interventions, which is likely scattered across several academic sources and ‘grey’ literature sources (i.e., non‐commercial). This EGM serves an important role in consolidating the research evidence in this field, a key purpose of systematic mapping approaches (Gough et al. 2017; Snilstveit et al. 2016). Further, there is limited pooled knowledge about the implementation or effectiveness of these types of interventions, or understanding of the experiences of those who have been involved in the interventions. Therefore, this EGM serves to identify interventions and pockets of research that are fruitful areas for further in‐depth analysis and research synthesis. It serves as a valuable tool in the process of engaging with stakeholders and identifying priorities for further analysis (Gough et al. 2017). Such analysis, together with the EGM, provides a useful resource for practitioners and policy‐makers who may be interested in relevant evidence to inform intervention design and implementation (White et al. 2018).

Concurrently, this EGM serves to highlight the gaps in research knowledge about ISS interventions. This is important for two reasons. First, acknowledging the limitations of the evidence base can serve to pinpoint which interventions are operating in an ‘evidence free’ area (White et al. 2018). Without adequate research knowledge, it may be difficult for practitioners or policy makers to determine how/whether interventions are facilitating appropriate and helpful forms of social support rather than negative responses (which have been associated with adverse outcomes for victim‐survivors, see Femi‐Ajao et al. 2018; Rizo and Macy 2011; Sylaska and Edwards 2014). Second, the EGM highlights important research gaps that can be used to inform the strategic commissioning of new primary research in the field (White et al. 2018).

### 2.4 Existing EGMs and/or Relevant Systematic Reviews

To our knowledge, there has been no attempt to map the evidence on ISS interventions but there have been systematic reviews focusing on relevant interventions. There are a few systematic reviews that evaluate informal support interventions alongside other types of intervention. For example, support groups for victim‐survivors, in person or online, are included alongside a range of other interventions in systematic reviews of tertiary interventions in DVA (Anderson et al. 2019; Rempel et al. 2018; Trabold et al. 2018). It is, therefore, not possible to isolate the efforts of informal mechanisms of support, as distinct from other interventions, in these reviews. To our knowledge, there are two reviews that have focused solely on research of peer support groups. These reviews report positive but tentative impacts for survivors of abuse (Sullivan 2012; Konya et al. 2020). Only one systematic review examines the effectiveness of a wider set of interventions pertaining specifically to ISS (Ogbe et al. 2020). This review evaluated DVA interventions that aimed to improve access to social support and mental health outcomes of victim‐survivors, reporting positive impacts of advocacy interventions with strong community linkages. The review included a range of interventions that facilitated informal support for DVA survivors, often alongside or through formal mechanisms of support (such as nurses, psychologists or in shelter contexts).

This EGM builds on, and is distinct from, existing systematic reviews in the following ways. First, the scope of the EGM is broader than reviews that focus on one type of informal support intervention (such as Sullivan 2012; Konya et al. 2020) but narrower than reviews that include multiple interventions, which may also include an element of formal support (Ogbe et al. 2020). This EGM examines different interventions aimed at enhancing, promoting or creating ISS. Interventions that also include an element of professional/formal social support (e.g., practitioner advice provided in an online forum or support group) are excluded in an attempt to pinpoint research on interventions that provide informal mechanisms of support. Second, the EGM takes a broader perspective by including any interventions that mediate the provision of informal support rather than only addressing interventions that improve access to such support for DVA victim‐survivors (as per Ogbe et al. 2020 review). Interventions that target the nature and type of support or address informal supporters rather than victim‐survivors, for example, are included.

## 3 Objectives

The EGM aims to identify and describe all available empirical research on interventions aiming to create or enhance informal support for victim‐survivors of DVA.

The specific objectives of the EGM are:

- Establish the nature and extent of the primary empirical evidence, reporting qualitative or quantitative data, on ISS interventions.
- Identify interventions and clusters of evidence suitable for systematic review/evidence synthesis.
- Identify gaps in the evidence on ISS interventions.

## 4 Methods

The protocol for this EGM was published with the Campbell Collaboration: Schucan Bird et al. (2022).

### 4.1 EGM: Definition and Purpose

EGMs describe the breadth, purpose and extent of research activity within a given field and/or focus (Gough et al. 2017). Such maps utilise systematic review methods to identify, describe and represent the existing evidence base, serving to highlight the availability and characteristics of such research (Snilstveit et al. 2016). This EGM undertook the following process, informed by accepted standards of EGM conduct (White et al. 2018): Developed and established a framework for understanding ISS interventions (what they are and how they may differ), identified and described all available empirical research on such interventions, developed a visual representation of the EGM and analysed the findings to draw inferences for research, policy and practice audiences. Stakeholder engagement was embedded in this process.

The EGM was a collaboration between university‐based academics and specialist DVA researchers from a domestic abuse organisation. This collaboration was integrated into the design and budget of the project from the outset, to enable meaningful contribution from all parties, build capacity in systematic reviews and create valuable outputs.

### 4.2 Framework Development and Scope

Whilst the literature recognises that ISS interventions vary according to ‘the types and sources of social support they seek to mobilize’ (Budde and Schene 2004, 344), there is no overarching framework for ISS interventions in the field of DVA (Nolet et al. 2020). A framework for the interventions was therefore developed for this EGM. This framework was initially developed by KSB through interaction with two sets of literature: (1) studies of ISS interventions for DVA and (2) research that offered empirical or theoretical contributions to understanding the role played by ISS interventions in leading to change for victim‐survivors. The resulting framework was refined in collaboration with NS and CR. The framework was presented to and reviewed by the Advisory Group (see ‘Stakeholder engagement’ below). Subsequently, the conceptual framework was mapped onto the ecological framework of DVA (Heise 1998). This serves as an analytic tool for pinpointing the multiple and intersecting causes of DVA, set across four different levels: indiVidual, relationship, community and societal. The ecological framework is used to differentiate DVA interventions, recognising that they may target single or multiple levels/factors (World Health Organisation 2021). More broadly, the ecological model is recognised as a valuable tool for drawing inferences from systematic review‐level findings and informing recommendations for policy/practice about DVA (Shorey 2023; Weeks and LeBlanc 2011).

**Figure 1:**
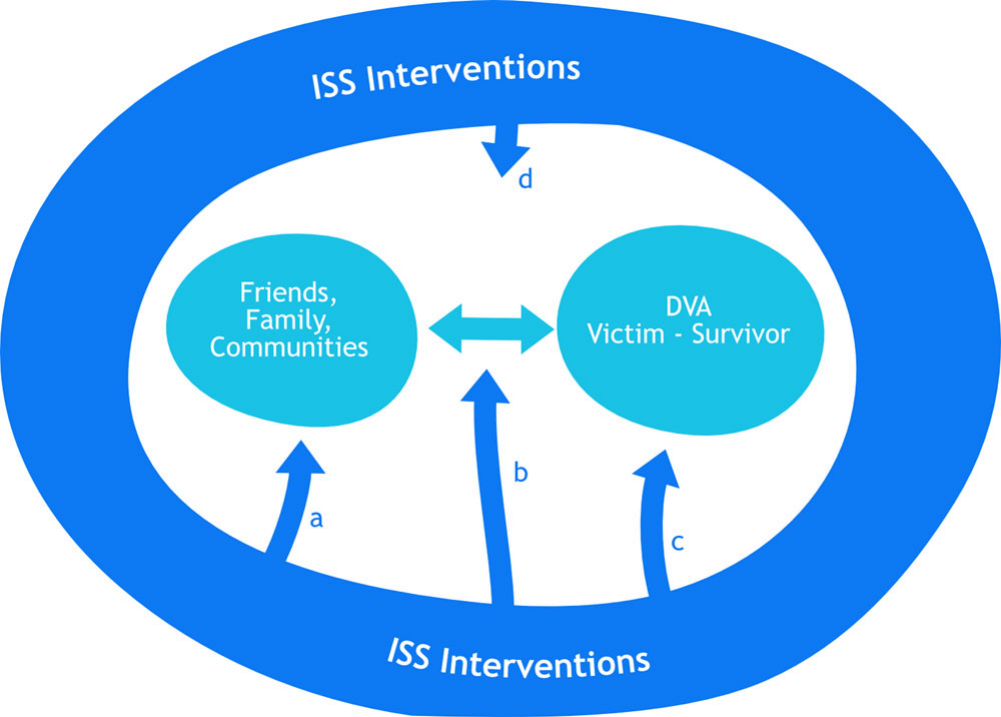
Types of informal social support intervention.

Figure 1 presents a visual overview of a framework for understanding the nature and range of interventions that aim to create, enhance or facilitate ISS for DVA victim‐survivors. The relationship between the DVA victim‐survivor and informal supporters (friends, family members and communities) is located at the centre of the Figure. ISS Interventions, represented by the darker arrows, target four different aspects of the informal support relationship (as indicated by a, b, c and d). To be considered an ISS intervention, and be included in the EGM, interventions (or one component of them) needed to *explicitly aim* to enhance/promote informal support. The framework includes interventions that are tailored towards the informal network as the *provider* of support (a), interventions that focus on how social support is provided and the *relationship* between the victim‐survivor and informal supporter (b), interventions that focus on the *victim‐survivors and their ability/resources* to engage with, and utilise, informal support (c), and interventions that address the *wider community* within which ISS is provided (d). The scope of ISS interventions varies, and they can include one or more of these (potentially overlapping) foci. This framework differentiates interventions based on two key elements: (1) the population targeted by the intervention. Whether the ISS intervention is tailored towards victim‐survivors (c), individuals or groups with specified informal supporter role (a, b), or the wider community/population at large who constitute an untapped source of informal support (d); (2) the primary level of the ecological model targeted by the intervention (i.e., whether tailored towards individual, relationship, community or societal factors) (Heise 1998; World Health Organisation 2021) (Figure 1).

(a) ISS interventions targeting the informal supporter, and individual/personal factors that shape their provision of support. Interventions seek to create new forms of informal support by galvanising existing social support networks (enhancing recognition and understanding of DVA by existing friends, relatives, neighbours or colleagues) and/or mobilising potential supporters (creating new relationships or networks of informal support). Interventions targeting the provider of informal support are tailored towards specific types of supporters, such as friends or colleagues, within the scope of the social or informal networks of victim‐survivors. These supporters may or may not already have a relationship with the victim‐survivor. These interventions target individual or personal level factors (Heise 1998) that shape the behaviours of the current/potential informal supporter. Such factors may include an individual's knowledge of DVA or seek to utilise their personal history/exposure to DVA. These types of intervention could include the training of potential informal social supporters (such as clergy in Choi et al. 2018 or peers in Tutty et al. 2016) to improve their knowledge and understanding of DVA.

(b) ISS interventions that shape the quality of support and/or relationship between informal supporters and DVA victim‐survivors. These interventions typically target individuals or groups who already have an informal relationship with the victim‐survivor. Such interventions are tailored towards interpersonal relationships and focus on the interactions between victim‐survivors and their wider networks (World Health Organisation 2021). Interventions could target the nature of communications between victim‐survivor and their informal networks, for example, and so include the moderation of exchanges between victim‐survivors and supporters in an online forum (e.g., Berg 2015).

(c) ISS interventions that primarily focus on the victim‐survivors and their ability to engage with, and utilise, informal support. Such interventions are tailored towards the victim‐survivor and their capacity to receive and utilise support from their informal and social networks. As such, these interventions are primarily targeted at the individual and/or personal level factors (Heise 1998) that shape victim‐survivors' interactions with informal networks. Such factors could include an individual's emotional capacity to engage with informal supporters or the make‐up of their personal networks. These types of intervention include, for example, practitioners assisting victim‐survivors to re‐engage existing networks or develop new forms of social support (Goodman et al. 2015).

(d) ISS interventions that target the community and/or society within which the informal support takes place. Communities are both physical places/spaces and forms of social organisation within which informal support operates (Mancini et al. 2006). These types of intervention aim to change the social environment in which informal support is provided by targeting community and societal‐level factors that facilitate or inhibit the provision of positive support. Such factors include, for example, norms and values about marriage, acceptability of abuse, and social cohesion (Heise 1998; World Health Organisation 2021; Wright et al. 2017). These interventions are not tailored towards a particular type of informal supporter (such as colleagues or relatives) but focus on providing resources and opportunities to the community at large (‘population‐level approach’) (Walsh et al. 2023). Therefore, these individuals and groups do not typically have a pre‐existing relationship with the victim‐survivor. Examples of intervention include small, specialised community projects to facilitate social support, for example, local fora for DVA (Wilcox 2000) or wider initiatives that deliver education and raise awareness about violence and abuse in a community setting to improve support for victims (e.g., community co‐ordinators delivering presentations in key community spaces, such as described in Flanigan 2011).

### 4.3 Stakeholder Engagement

Early and continuing engagement with stakeholders is critical for rapid reviews and EGMs to ensure relevance and actionable outputs (Tricco et al. 2017). An Advisory Group was created at the outset with members nominated and recruited through the networks of the researchers and their respective organisations. Six stakeholders were selected for the Advisory Group. This included: two people with expertise by lived experience, who were recruited from the domestic abuse organisation's group of ‘Pioneers’ (a group of survivors of abuse), two representatives of frontline DA services (Kate Lawrence Home‐Start East Sussex; Mollin Delve, PHOEBE) who had previously worked with the domestic abuse organisation and indicated an interest in informal support networks, a researcher with expertise in informal supporters (Dr. Alison Gregory, former University of Bristol) and a policy/practice representative (Dr. Andy Myhill, College of Policing). The composition of the Advisory group aimed to ensure the representation of different perspectives based on ethnicity and geographical locations of the stakeholders/groups they were representing.

Two meetings were held to co‐ordinate stakeholder engagement in the EGM. The first meeting of the Advisory Group established the scope of the review and took place online in May 2021. Discussions and polling were used to define the framework for categorising the interventions (types of ISS interventions) and the inclusion criteria (type of research, population groups and outcomes that were deemed eligible for inclusion). The Advisory Group meeting led to the broadening of the scope of the EGM to include informal supporters as part of the study population (rather than only victim‐survivors), victim‐survivors who live in shelters/refuges, and to include studies that focus on victim‐survivors that have left the abusive relationship (whether in the short or longer term). The Advisory Group emphasised the importance of including studies that potentially included an element of professional involvement as facilitating support.

The initial meeting also provided information that fed into the search sources and data extraction methods. The Advisory Group stressed the importance of grey literature in this field. The Advisory Group highlighted the importance of capturing information from the studies, where available, of the informal supporters' prior lived experience of abuse, demographic, cultural and social characteristics, and training. All study outcomes were of interest for the EGM, with the project led by Howarth et al. offering a helpful framework for classifying outcome measures (see Powell et al. 2022).

The second meeting took place online in July 2021. The Advisory Group reviewed the initial findings of the EGM and identified priority areas for further analysis and evidence synthesis (see Schucan Bird et al. 2023a and Schucan Bird et al. 2024 for findings from the subsequent evidence syntheses).

### 4.4 Ethical Considerations

The EGM gained ethical approval from the IOE Research Ethics Committee and aimed to embed ethical practices throughout the review. Key considerations included gaining informed consent from the Advisory group for involvement in the EGM and associated outputs, ensuring diverse perspectives were represented in the review (via the Advisory group, incorporating ‘grey literature’, and extracting data on key cultural and demographic variables) and embedding collaborative working methods in the review processes. For a reflexive discussion of the ethical implications associated with this EGM, see Schucan Bird et al. (2023b).

### 4.5 Conceptual Framework: How ISS Interventions Are Expected to Work

This section draws on existing research evidence to build an understanding of how ISS interventions might lead to positive outcomes for victim‐survivors of DVA. In 2004, Budde and Schene (2004, 347) set out an agenda for evaluating ISS interventions, recognising that ‘we are in the early stages of learning how ISS interventions can contribute to preventing… domestic violence’. Whilst empirical work on informal support has grown, few studies have explored the role played by ISS interventions, alongside wider DVA service provision or formal agencies, in contributing to social, inter‐ and intra‐personal changes that lead to improvements for DVA victim‐survivors (Goodman et al. 2015; Sullivan 2017). All ISS interventions are expected to work by creating or enhancing informal support that is considered helpful by victim‐survivors, which can influence the recovery of the victim‐survivor (Klein 2012). The literature explains that victim‐survivors identify both negative and positive responses to disclosure. Studies define the provision of emotional support, advice and/or practical support as positive reactions to disclosures of abuse. Informal supporters who listen and allow the victim‐survivor to talk about the abuse are also helpful (Goodman and Smyth 2011; Nolet et al. 2020; Sylaska and Edwards 2014). In contrast, victim‐survivors identify negative responses as refusal of the problem by expressing doubt, blaming the victim or withdrawing support (Nolet et al. 2020; Sylaska and Edwards 2014). There is a body of literature that associates helpful responses with positive outcomes for the victim‐survivor, including improvements in quality of life, self‐esteem, autonomy and mental health (Beeble et al. 2009; Dworkin et al. 2019; Levendosky et al. 2004; Nolet et al. 2020; Sylaska and Edwards 2014) and promotion of help‐seeking behaviours (Liang et al. 2005). Therefore, ISS interventions are expected to work by harnessing and promoting helpful responses to victim‐survivors from current or potential informal networks.

This section considers how each type of intervention, as described and visualised above (see Figure 1), is expected to lead to improved outcomes for victim‐survivors. This provided the framework, akin to a ‘framework synthesis’ approach to systematic reviewing (Oliver et al. 2015; Thomas et al. 2017), that guided the EGM. This meant that key steps in the process such as defining the inclusion criteria, and the coding of studies were aligned with the following conceptual framework.

(a) ISS interventions targeting the provider of informal support.

Interventions tailored towards the informal supporter target individual‐level factors that predispose them to respond positively to victim‐survivors (Edwards and Dardis 2020). These include individual characteristics of the informal supporter, such as their gender, cultural background and/or prior experience of DVA (Edwards and Dardis 2020; Sylaska and Edwards 2014; Ullman 2021). Therefore, interventions may aim to stimulate support from those individuals who are more likely to offer sympathetic responses, such as women and/or those who have a personal history of DVA (Beeble et al. 2008). Individuals with a history of victimisation, for example, are likely to have a greater understanding of DVA and empathy for the victim‐survivor, and so better recognise their support needs (Ullman 2021). ISS interventions may seek to harness the responses of individuals/groups who are predisposed to providing helpful responses to victim‐survivors.

Alternatively, interventions may target individual‐level factors that are associated with less positive responses to victim‐survivors such as lack of knowledge of DVA or attitudes condoning abuse (Edwards and Dardis 2020). Research highlights that a lack of understanding of DVA hinders an individual's ability to identify abuse and respond in a positive way (Latta and Goodman 2011), so interventions may seek to tackle individual deficits in knowledge and chAllenge myths about DVA. These types of interventions may include elements of education, training and/or information tailored to their role as an informal supporter. Such interventions may target, for example, an individual's gendered assumptions that blame victim‐survivors or deem them unworthy of sympathy and so shape informal responses to female victim‐survivors (Mwatsiya and Rasool 2021; Rivera‐Cuadrado 2020). Indeed, it is widely recognised that DVA interventions need to raise awareness and improve understanding of domestic violence (Hester and Westmarland 2005). Changes in the knowledge and attitudes of informal supporters are expected to improve recognition of abuse and generate helpful responses to victim‐survivors (Edwards and Dardis 2020; Flecha 2021). In turn, this improved response is expected to lead to positive outcomes associated with helpful informal support.

(b) ISS interventions that shape the quality of support and/or relationship between informal supporters and DVA victim‐survivor.

Interventions tailored towards relational factors include the type of personal relationship between the informal supporter and victim‐survivor, and the nature of interactions between them.

Evidence suggests that the type of informal supporter, for example, whether a friend or family member, and the length/quality of the relationship with the victim‐survivor influences the nature of the informal response (Edwards and Dardis 2020; Rivera‐Cuadrado 2020; Sylaska and Edwards 2014). For example, close female friendships are deemed important sources of support for young women and frequent social contact is associated with more positive reactions to disclosures of abuse (McKenzie et al. 2020; Rivera‐Cuadrado 2020). Concurrently, close male family members, especially fathers, are less likely to serve as informal supporters (Sylaska and Edwards 2014). Interventions may therefore seek to harness relationships that are more likely to provide helpful support, tailored towards primary networks that are associated with more positive social reactions (Arón and Lorion 2003; Edwards and Dardis 2020; França et al. 2018). Interventions may seek to shape the nature and quality of the interactions between victim‐survivor and informal networks to maximise this support. Research highlights that informal supporters do not know how to respond appropriately to disclosures of violence and abuse (Gregory et al. 2017; McKenzie et al. 2020). To address this, interventions may promote interpersonal interactions, rooted in empathy and acceptance (Edwards and Dardis 2020), which are associated with better support and improved outcomes (Ullman 2021).

(c) ISS interventions that focus on victim‐survivors' ability to engage with, and utilise, informal support.

Social isolation is key to understanding DVA (Lanier and Maume 2009) and can constitute a core element of abuse (Flecha 2021; Vidu et al. 2021). The ongoing process of social isolation (Nolet et al. 2020) means that victim‐survivors are unable to identify anyone, apart from their current or former partner, who they can turn to for help (Lanier and Maume 2009). This impedes the abilities of victim‐survivors to seek and receive support from both informal and formal sources (Vidu et al. 2021). Social isolation interacts with wider sociocultural factors such as gendered norms (e.g., expectations about women's role in a family) that may render women particularly vulnerable to the isolating impacts of abuse (Lanier and Maume 2009). ISS interventions that target victim‐survivor's ability to engage with social networks are therefore critical for enabling individuals to access and receive helpful forms of support. Evidence suggests that abusive relationships impact the size, quality and strength of a victim‐survivor's social network (Katerndahl et al. 2012; Zapor et al. 2015). The size of a social network may initially expand after leaving an abusive partner but contract in the longer term (Nolet et al. 2020), and the victim‐survivor may redefine their social relationships in the process (Hydén 2015). Similarly, the quality of social networks changes over time with research highlighting that negative relationships with social supporters dominate the abusive and break‐up stages but shift towards more positive relationships in the longer term (Nolet et al. 2020). This means that interventions can employ several practices/activities to maximise opportunities to develop positive relationships and sources of support over time (Goodman et al. 2015; Nolet et al. 2020). Interventions that enhance informal support can serve to bolster victim‐survivors' well‐being and mediate the impact of abuse on several outcomes (Beeble et al. 2009; Goodman et al. 2005). Interventions that interrupt or target social isolation are also expected to support help‐seeking, serve as a protective factor against revictimisation and contribute to the process of change and long‐term recovery (Beeble et al. 2009; Goodman et al. 2005; Melgar et al. 2021; Zapor et al. 2015).

(d) ISS interventions that target the community and/or society within which the informal support takes place.

There are multiple layers of social support and networks for victim‐survivors, with community organisations, neighbours and community members having an important role to play in identifying and responding to violence and abuse (Arón and Lorion 2003). Research identifies the growing importance of interventions that focus on community capacity building and collective efficacy in responding to DVA (Edwards et al. 2014; Sullivan et al. 2017). Such interventions enhance the community response to DVA by promoting social cohesion and improving collective understanding, confidence and skills to respond to abuse in the community. Interventions seek to create communities that ‘hold those who use violence accountable, promote justice, and that provide adequate resources and opportunities for all community members’ (Sullivan et al. 2017, 127). Evidence suggests that strengthening community bonds can build durable systems of support for victim‐survivors who are more likely to seek help from their informal networks if they hold a sense of social belonging (Barrett et al. 2020)

Interventions targeting communities also focus on the social, cultural and community norms that shape victim‐survivors' readiness to disclose, for example, family honour (Güler et al. 2023) and informal supporters' willingness to respond, for example, beliefs about the sanctity of marriage (Goodkind et al. 2003). Such interventions may aim to chAllenge the norms that inhibit responses to DVA to foster a social and physical environment that is conducive to positive support for victim‐survivors.

Where studies included multiple ISS interventions, multiple intervention types were coded.

### 4.6 Dimensions

This EGM included any empirical study (qualitative or quantitative) that included an intervention that had an explicit aim to enhance/promote informal support for victim‐survivors of domestic abuse. Further details of the types of study design, intervention and population groups are provided below.

#### 4.6.1 Types of Study Design

All study designs were included in the EGM to understand the nature and extent of the primary empirical evidence on ISS interventions. This ensured maximum identification and coverage of intervention research on informal support. To date, many domestic violence interventions that are well established as ways of responding to victim‐survivors (such as shelters and hotlines) do not have an underpinning evidence base. Some routine interventions have not been rigorously tested or formally evaluated (Bender 2017) and so intervention studies in the field of DVA are relatively sparse. Experimental studies are particularly lacking (Feder et al. 2011). The focus on ISS interventions which, by definition, sit outside formal agencies or services, means that rigorous evaluation is less likely to be mandated, and/or sufficiently funded. Indeed, existing systematic reviews of interventions delivered by the voluntary sector report a low number of studies, potentially explained by the lack of capacity and/or resources of this sector to carry out research (Konya et al. 2020). Therefore, this EGM was inclusive in terms of study design and eligible forms of research evidence.

For the methods of this EGM, this meant:

(1) Diverse study designs, reporting qualitative or quantitative data, were included. As outlined above, sources of evidence that evaluate ISS interventions are likely to be diverse in study design, method and type of data. Moreover, there is an established precedent for using qualitative and quantitative reports together in understanding the impacts of informal support interventions (Ogbe et al. 2020; Konya et al. 2020). Strict limits in terms of methodological approach were not applied because we know that intervention studies in the field of domestic violence face several challenges, including small sample sizes and reliance on pilot interventions (Trabold et al. 2018). Eligible studies therefore aimed to assess the impact/perception of the impact of the intervention against the outcomes stipulated below. Reports were excluded if they described a programme/intervention but did not provide any data (qualitative or quantitative) that served to evaluate the intervention (as outcomes or perceptions of outcomes).

The study designs of included studies were classified according to the categories outlined in Hong et al. (2018): Qualitative, Quantitative randomised controlled trials, Quantitative non‐randomised, Quantitative descriptive and Mixed Methods. Study design and type of data were captured by the Data Extraction tool (see Supporting Information 2).

Eligible systematic reviews were not directly included in the EGM but used as a search source to identify primary empirical studies.

(2) Grey literature was included.

ISS interventions are likely to be delivered by and/or involve voluntary sector organisations, workplaces and/or wider community organisations. Research associated with such interventions is therefore likely to be reported in ‘grey’ literature sources alongside or instead of traditional academic channels. Grey literature includes ‘that which is produced on all levels of government, academics, business and industry in print and electronic formats, but which is not controlled by commercial publishers’ (cited in Tyndall 2010). This EGM included dissertations, conference papers and research reports published by organisations in the field of DVA.

Evidence was only included if the full text of the study was published in English to meet the rapid imperative of this project. Funded as part of the UK Research and Innovation (UKRI) response to the Covid‐19 pandemic, this project used streamlined methods to complete the EGM in a relatively short period of time to satisfy extant need. Limiting the selection by language is one such common method (Tricco et al. 2015).

Ongoing studies were excluded if there was no clear completion data/uncertainty about completion.

#### 4.6.2 Types of Intervention

Eligible studies needed to include an ISS intervention targeting DVA. Such interventions are defined as ‘Systematic activities designed to change the existing quality, level or function of an individual's personal social network or to create new networks and relationships’ (Budde and Schene 2004, 342). To be eligible, the intervention needed to fit within the parameters of the following criteria.

*Type of ISS intervention*: The EGM recognises that ISS interventions are diverse, varying in their aims and nature. Interventions aim to create or enhance ISS by targeting the providers of the ISS, the victim‐survivor or the relationship between them. Interventions can also aim to change the wider community/society within which ISS is provided (as outlined in the Conceptual Framework above).

Whilst a wide range of interventions may have an indirect effect on informal support for victim‐survivors of abuse, this EGM was only interested in those interventions that specifically target and promote informal support. Therefore, to be included, interventions (or one component of them) needed to have an *explicit aim* to enhance/promote informal support. Interventions may also have provided support to victim‐survivors, but this was not an explicit criterion for selection. For example:

- Support groups for DVA victim‐survivors are only included if/when the intervention explicitly aims to enhance informal support.
- Advocacy interventions are only included if they include an explicit aim to support the victim‐survivor to build or enhance their informal social networks.
- Community‐focused interventions are only included when they are tailored towards the informal support relationship (e.g., training community members as advocates) rather than general community‐focused DVA interventions (e.g., general DVA awareness‐raising campaigns that are not explicitly linked to informal support).

*Provider of ISS*: ISS interventions enable the provision of support to victim‐survivors from their friends, colleagues, neighbours or community members, current non‐abusive partners or any family member. To do so, an intervention may serve to bolster the provision of *direct* support from victim‐survivors' existing networks and/or involve external actors in the facilitation of informal support seeking. This EGM recognises the growing role of professions in facilitating informal support (e.g., Goodman et al. 2015) and eligible interventions may have included a role for professionals/practitioners in *facilitating* the victim‐survivor's ability to engage with their informal social supporters, for example, supporting the victim‐survivor to reach out and/or broaden their social networks. Interventions were excluded if social support was directly provided by formal sources/professionals. This refers to the support provided by professionals and/or practitioners working in services provided by the state, non‐governmental organisations/third sector and the legal system−police, domestic abuse professionals, shelters, support workers and counselling (Kelly et al. 1996). Similarly, this EGM recognised that eligible interventions may have included payment for volunteers or professionals in their role as facilitating/promoting ISS.

#### 4.6.3 Types of Population

The study population needed to include and report data on *(1) Victim‐survivors of DVA* AND/OR their *(2) Informal supporters*

1. *Victim‐survivors of DVA* (Any adults or young people who are/have been experiencing violence and abuse in a current or former intimate relationship). This EGM focused on individuals who had or were experiencing intimate partner violence and abuse, as opposed to other forms of family violence and abuse (e.g., sibling abuse or child‐to‐parent abuse). The focus on abuse in an intimate relationship is common in systematic reviews of interventions to address DVA (e.g., Anderson et al. 2019; Rivas et al. 2016), including reviews that have focused specifically on informal support (e.g., Gregory et al. 2017; Ogbe et al. 2020). The definition of DVA was based on the UK Domestic Abuse Act (2021): Abusive behaviour includes a single incident or course of conduct of physical, sexual, economic, psychological, emotional or other abuse, and/or violent, threatening, controlling or coercive behaviour. There were no restrictions on age, nationality or sex/gender of the population. An exception to this, however, was that children who witnessed DVA (recognised as victims by the Domestic Abuse Act 2021) were not an eligible population group in this review. This was because the abusive relationship is not between intimate partners. This criterion means that there was a natural age restriction to the study.
2. *Informal supporters* refer to friends, colleagues, neighbours or community members, current non‐abusive partners and any family member (including step‐family, non‐blood relatives and family‐in‐law) (developed from the Gregory et al. 2017 definition).

To be included in the EGM, studies with a mixed sample (e.g., formal supporters such as practitioners/professionals and informal supporters), needed to report data separately for each population group (so findings for victim/survivors and/or informal supporters could be isolated).

#### 4.6.4 Types of Outcomes

This EGM did not use outcomes as eligibility criteria. In line with the Conceptual framework, the EGM mapped the evidence against a broad range of outcome categories. These encompassed outcomes measured across the socio‐ecological framework of DVA. At the level of individuals (i.e., measuring and representing attributes of individuals such as attitudes or knowledge of victim‐survivors or informal supporters), relationships (i.e., measuring the characteristics of a relationship between two or more individuals e.g., nature and quality of victim‐survivors' relationships with the social network) and community/societal (i.e., measuring attributes of a whole population group and representing activities across that community, for example, collective norms and values relating to DVA or responses to victim‐survivors from community members). Initial outcome domains were pre‐defined in the protocol as presented in Table 1. This illustrates how different forms of data (qualitative and quantitative) and data instruments were coded against each outcome domain. New domains and categories were added to the outcomes as and when they emerged from included studies. Qualitative outcomes were derived from different levels of data, including quotes from participants (first‐order data), themes determined by authors of the primary study (second‐order data), or interpretation by review authors/coders (third‐order data). While the different order data have different levels of reliability, this is a recognised approach for meta‐ethnographic reviews of qualitative data (e.g., Sandelowski and Barroso 2006).

**Table 1:** Outcome categories.

Category	Definition	Example quantitative instruments	Example qualitative data (1^st^, 2^nd^ or 3^rd^ order)
Cognitive	Knowledge or attitudes about DVA, including awareness of support and resources.	Domestic Violence Myths Acceptance Scale (DVMAS)	‘Program participants declared that the sessions they found most helpful involved content on: (a) safety planning, (b) community resources to address and obtain help with IPV, (c) information about the cycle of violence, and (d) information about the effects of IPV on women and children’. (2nd order data in Macy et al. (2012), p. 459)
Behavioural	Any behaviour or action including formal help‐seeking by victim‐survivor or provision of support by informal supporter	Decisional Conflict Scale	‘I'm still taking the help she gave me and using it on my own’ (1st order data in Allen et al., 2012, p. 12) Women who formerly faced gender‐based violence in silence began to call for help (3rd order data, Schuler et al. (2011))
Social network	The structure (size, density, composition), function (provision of support/response) or dynamics (relationship) of the victim‐survivor's social support network	The Interpersonal Support Self Evaluation List (ISEL)	‘Rejoining the community’ theme (2nd order data in Allen and Wozniak (2010), p. 50)
Violence and Abuse	Experiences of violence and abuse	Severity of Violence Against Women Scale (SVWAS)	‘It's got a lot less toxic, basically’ (1st order data in Zakheim (2009), p. 129)
Characteristics of relationship between victim‐survivor and perpetrator	Characteristics of the relationship, e.g. emotional involvement, intimacy, love, communication, decision‐making	The Attitudes Towards Marriage and the Family Scale	‘A woman during her interview said that she used to obey her husband and his family member's orders even for small issues like when to eat’ (2nd order data in Mahapatro & Singh, 2020, p. 287)
Mental health	Clinical/diagnosis of mental health such as depression, PTSD, anxiety.	PTSD Checklist	Women used coping strategies that served to protect their mental health (3rd order data in Mahapatro & Singh, 2020)
Psycho‐Social	Quality of life, well‐being and self‐efficacy	Domestic Violence Self‐Efficacy (DVSE) instrument	‘…it just makes me feel better and just gives me strength each time’ (1st order data in Taylor, (2000), p. 521)
Physical Health	Physical Health	The Health Screening Questionnaire (HSQ)	

Core outcome sets for DVA developed by Powell (2022), recommended by the Advisory Group, did not inform the data extraction tools because the work of Powell and colleagues had not yet been completed. Therefore, the outcomes covered in this EGM have been compared with the core outcome sets in Section 6 below.

### 4.7 Search Methods and Sources

**Figure 2:**
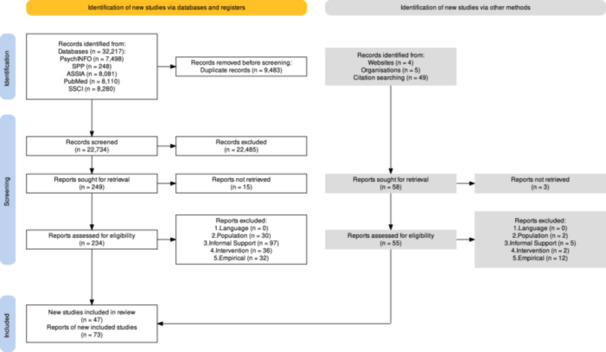
Flow of studies in the EGM (PRISMA diagram).

The search strategy sought to explicitly target social science literature, rather than primarily health research (as per Ogbe et al. 2020), including dedicated sources of grey literature and without limits on the publication date (as per Konya et al. 2020). To ensure comprehensive coverage of academic and grey literature sources, a multi‐stranded search approach was undertaken. As illustrated in Figure 2, new studies were identified via two channels: (1) Databases and registers: bibliographic databases, specialist DVA databases, policy‐orientated databases and systematic review databases; (2) Handsearching and other methods: manually screening documents on websites of DVA organisations. Citation checking of relevant systematic reviews and key primary studies was also undertaken. Key authors were also contacted.

1. *Databases and registers*
  Seeking to identify all types of study design and forms of data, the search strings used for database searching were intentionally broad and based on two core concepts: domestic violence and abuse AND informal social support. The search terms used in the electronic databases were informed by those implemented in previous reviews (Ogbe et al. 2020; Gregory et al. 2017). Terms associated with each concept were identified and used to generate the search strings.
  The searching and screening processes were carried out in two phases. The bibliographic database searching was completed on 31st October 2022 and screening of the results was undertaken. Subsequently, specialist database searching (including ‘Policy orientated databases’, ‘Systematic review databases’ and ‘Specialist DVA databases’) was completed on 10th July 2023. Further details for each source are outlined below.
  Bibliographic databases: Five sources were used to search across disciplines relevant to DVA. A search string was developed based on terms used in similar reviews (Ogbe et al. 2020; Gregory et al. 2017). Databases include APA PsycINFO via Ovid (search terms specified in Supporting Information S1), Social Policy and Practice via Ovid (search terms in Supporting Information 1), ASSIA via ProQuest (search terms specified in Supporting Information 1), PubMed (search terms specified in Supporting Information 1) and Social Science Citation Index via Web of Science (search terms specified in Supporting Information 1).
  Policy‐orientated databases: European Commission Regional Policy Projects Database and Horizon Project results platform using terms ‘domestic violence’ or ‘domestic abuse’, European Commission CORDIS database (search terms specified in Supporting Information 1 ‘Search terms for systematic review and policy‐orientated databases’), World Health Organisation Publications with Health topic ‘Violence against women’.
  Systematic review databases: Social Systems Evidence, Campbell Collaboration (search terms specified in Supporting Information 1 ‘Search terms for systematic review and policy‐orientated databases’).
  Specialist DVA databases: National Resource Centre on Domestic Violence, Anrows Library UN Women Virtual Knowledge Centre to End Violence against Women and Girls (search terms specified in Supporting Information 1 ‘Search terms for DVA specialist databases and websites’).
2. *Handsearching and other methods*
  Handsearching websites of DVA organisations: The websites of the following organisations were manually searched by examining the documents listed on the ‘research’ or ‘evaluation’ sections of the website and/or using the search function (search terms, where applicable, specified in Supporting Information 1 ‘Search terms for DVA specialist databases and websites’): SafeLives, Women's Aid, Imkaan, AVA, Respect, Standing Together, Galop, Surviving Economic Abuse, Refuge, Solace, Mankind, Sagesse, Domestic Violence Resource Centre Victoria, Loved Ones of Domestic Abuse Survivors, The Empowerment Network, Bambuuu, Bromley Well.
  Citation chasing: (1) Backward citation chasing was employed to identify studies/intervention reports referenced by relevant systematic reviews. The reference lists of 22 systematic reviews (identified during the screening of records from the database search) were checked, with studies in the reference list screened on the title. Backward citation techniques were also used to identify potentially relevant studies referenced by included studies, also screened on title. (2) Forward citation chasing was used to identify potentially relevant studies that had cited key excluded studies; Google Scholar was used to identify studies that had cited excluded studies that were highly relevant but did not report empirical data. Potentially relevant studies were screened on title and abstract.
  Contacting authors, stakeholders and networks: Members of the advisory group were asked for recommendations for studies, reports and/or evaluations. A limited number of authors in the field of ISS interventions were also contacted to seek further potentially eligible studies. Email requests were sent to 10 authors and requests were included in newsletters to wider networks: The Violence, Abuse, and Mental Health Network (VAHMN) and SafeLives' Leading Lights services.

### 4.8 Analysis and Presentation

#### 4.8.1 Report Structure

The results of the EGM provide a descriptive overview of the nature and extent of the primary empirical research on ISS interventions. This is structured according to the main types of intervention (type, setting, provider of social support) study population (victim‐survivors, informal supporters, practitioners and/or communities, geography), study design and outcomes (specified in Table 3).

The report describes and analyses the spread and concentration of the evidence across the four types of intervention, and how these interventions map onto the socio‐ecological model of DVA (Heise 1998; World Health Organisation 2021). It explores the type of outcomes that have been reported, including a consideration of the outcomes reported for victim‐survivors by their gender, ethnicity, age, exposure to abuse and migration status. The report highlights evidence trends and the key gaps/limitations of the current evidence base. Policy, practice and research implications are considered.

Figures include the conceptual framework and flow of studies through the EGM. Tables summarise included studies, by intervention type.

#### 4.8.2 Filters for Presentation

The findings of the EGM are presented as a matrix of intervention type and outcomes. Additional filters include geographical region, study population (victim‐survivors or informal social supporters) and characteristics of informal support (setting, provider). The size of the bubble reflects the number of studies and the colour of the bubbles represents the study design.

### 4.9 Dependency

Each item on the map represents a single study. Where multiple reports exist for the same study, a main report is identified and relevant reports are used for descriptive purposes.

### 4.10 Data Collection and Analysis

#### 4.10.1 Screening and Study Selection

To develop a shared understanding of the eligibility criteria and screening process, a team of four reviewers initially screened a sample of titles and abstracts in pairs. Decisions were discussed and discrepancies between pairs were resolved by a third reviewer until a high level of consistency was reached (and inclusion/exclusion criteria were sufficiently clear). Individual reviewers were then screened independently, with a process built in for identifying studies that required the judgement of a second reviewer. A sample of 45 studies included in the title and abstract were then screened on full text by the four reviewers. Individual reviewers then worked independently, with a process built in for identifying studies that required the judgement of a second reviewer. Decisions on complex studies were discussed by all four reviewers.

All references identified in the search were imported and screened using EPPI Reviewer software (Thomas et al. 2023).

#### 4.10.2 Data Extraction and Management

A data extraction tool was developed and piloted by four members of the team. The EGM extracted data on: Study Population, Study, Intervention and Outcomes. For the complete tool see Supporting Information 2. This tool integrated the recommendations made by the Advisory Group.

The EGM aimed to identify and describe the evidence base rather than reporting the findings of individual studies or assessing methodological quality. Therefore, in alignment with standards of conduct for EGMs, the quality of primary studies was not assessed (Snilstveit et al. 2016; White et al. 2018) but study designs were coded and reported.

All studies included in full text and subsequently in the EGM were coded by two independent reviewers. Coding decisions were discussed and agreed upon with a third reviewer serving as moderator, where necessary.

EPPI Reviewer software was used for the process of data extraction, information management and production of the online evidence map.

#### 4.10.3 Tools for Assessing Risk of Bias/Study Quality of Included Reviews

Quality appraisal of primary studies or systematic reviews was not undertaken. Systematic reviews were not included in the EGM but are used as a source of primary studies.

### 4.11 Methods for Mapping

EPPI Reviewer Software was used for the entire EGM process.

## 5 Results

### 5.1 Description of Studies

#### 5.1.1 Results of the Search

The search of bibliographic databases yielded 32,217 records. After 9483 duplicates were removed, 22,734 items were screened on title and abstract. In total, 249 records from the database search were sought for retrieval, of which 234 were screened on full text. This resulted in 39 items, originally identified in the database search, being included in the EGM. Alongside the database searching, handsearching and other methods identified 58 potentially eligible items; 55 of these were assessed for eligibility and 34 studies were included. There was a total of 47 studies included in the EGM (and 26 secondary, linked reports). The flow of studies is presented in Figure 2, using Haddaway et al. (2022).

#### 5.1.2 Excluded Studies

A total of 216 reports were excluded at full text review. The main reason for exclusion was that the study did not focus on ISS (*n* = 102), followed by a lack of empirical data/methods (*n* = 44), not an intervention (*n* = 38), or the study population did not include victim‐survivors or informal supporters (*n* = 32). No reports were excluded on language in the full text screening phase (see Figure 2). Key excluded studies are listed in Table 2 together with the reason for exclusion.

**Table 2:** Characteristics of excluded studies (*n* = 69).

Item	Screening on full text
Ablaza et al. (2023)	EXCLUDE: Not Informal Social Support
Andrews (2021)	EXCLUDE: Not Informal Social Support
Armstead (2018)	EXCLUDE: Population (NOT victim‐survivors or Informal Supporters)
Aron (2003)	EXCLUDE: Not Empirical/ no data
Banister (2007)	EXCLUDE: Not Informal Social Support
Banks (2008)	EXCLUDE: Not Informal Social Support
Belknap (2010)	EXCLUDE: Not Informal Social Support
Bell (2015)	EXCLUDE: Population (NOT victim‐survivors or Informal Supporters)
Bhandari (2012)	EXCLUDE: Not intervention
Bloom (2016)	EXCLUDE: Population (NOT victim‐survivors or Informal Supporters)
Bradbury‐Jones (2017)	EXCLUDE: Not Informal Social Support
Brownell (2006)	EXCLUDE: Population (NOT victim‐survivors or Informal Supporters)
Bryant‐Davis (2013)	EXCLUDE: Population (NOT victim‐survivors or Informal Supporters)
Burnham (2022)	EXCLUDE: Not Informal Social Support
Campbell (1986)	EXCLUDE: Not Empirical/ no data
Choi (2019)	EXCLUDE: Not Informal Social Support
Clark (2018)	EXCLUDE: Not Informal Social Support
Clark (2020)	EXCLUDE: Not Informal Social Support
Clark (2021)	EXCLUDE: Not Informal Social Support
Cline (2022)	EXCLUDE: Population (NOT victim‐survivors or Informal Supporters)
Constantino (2015)	EXCLUDE: Not Informal Social Support
Corwin (2020)	EXCLUDE: Population (NOT victim‐survivors or Informal Supporters)
Cuomo (2022)	EXCLUDE: Not intervention
DiVietro (2016)	EXCLUDE: Not Informal Social Support
Edwards (2021)	EXCLUDE: Population (NOT victim‐survivors or Informal Supporters)
Frohmann (2005)	EXCLUDE: Not Informal Social Support
Goodman (2016)	EXCLUDE: Population (NOT victim‐survivors or Informal Supporters)
Gregory (2021)	EXCLUDE: Population (NOT victim‐survivors or Informal Supporters)
Hailemariam (2022)	EXCLUDE: Not Empirical/ no data
Hansen (2014)	EXCLUDE: Not Informal Social Support
Hatcher (2020)	EXCLUDE: Not Informal Social Support
Hellman (2006)	EXCLUDE: Population (NOT victim‐survivors or Informal Supporters)
Honda (2022)	EXCLUDE: Population (NOT victim‐survivors or Informal Supporters)
Hurley (2007)	EXCLUDE: Not intervention
Inge (2009)	EXCLUDE: Not Empirical/ no data
Jones (2009)	EXCLUDE: Not Empirical/ no data
Kamimura (2013)	EXCLUDE: Not Informal Social Support
Kaminaga (2020)	EXCLUDE: Not intervention
Kohli (2015)	EXCLUDE: Not intervention
Kugel (2009)	EXCLUDE: Not intervention
Kyegombe (2022)	EXCLUDE: Population (NOT victim‐survivors or Informal Supporters)
Leitao (2021)	EXCLUDE: Not intervention
Lilleston (2018)	EXCLUDE: Population (NOT victim‐survivors or Informal Supporters)
Mannell (2018)	EXCLUDE: Not Empirical/ no data
Marin (2012)	EXCLUDE: Not intervention
Marrs (2013)	EXCLUDE: Not Informal Social Support
McCauley (2018)	EXCLUDE: Not Informal Social Support
Mcdonald (2011)	EXCLUDE: Not Informal Social Support
McFarlane (1997)	EXCLUDE: Not Informal Social Support
Miller (2014)	EXCLUDE: Not Informal Social Support
Nair (2020)	EXCLUDE: Not Informal Social Support
Naved (2021)	EXCLUDE: Not Informal Social Support
Nelson (2010)	EXCLUDE: Not Informal Social Support
Whiting et al. (2022)	EXCLUDE: Not intervention
Pennell (2021)	EXCLUDE: Population (NOT victim‐survivors or Informal Supporters)
Ragavan (2020)	EXCLUDE: Not Informal Social Support
Roberto (2013)	EXCLUDE: Not Empirical/ no data
Sarnquist (2021)	EXCLUDE: Not Informal Social Support
Serrata (2016)	EXCLUDE: Not Informal Social Support
Singleton (2019)	EXCLUDE: Not intervention
Stern (2018)	EXCLUDE: Population (NOT victim‐survivors or Informal Supporters)
Stevens (2015)	EXCLUDE: Not Informal Social Support
Taft (2009)	EXCLUDE: Not Empirical/ no data
Taylor (2019)	EXCLUDE: Not intervention
Watanabe et al. (2022)	EXCLUDE: Not Informal Social Support
Tiwari (2012)	EXCLUDE: Not Informal Social Support
Tiwari (2012)	EXCLUDE: Not Informal Social Support
Wagman (2018)	EXCLUDE: Not Informal Social Support
Whitaker (2007)	EXCLUDE: Not Empirical/ no data

Almost half of the studies were excluded because they did not focus on informal support. Ablaza et al. (2022) focused on ‘non‐specialist’ support but the associated services were delivered by both professionals and volunteers; the intervention in Choi et al. (2019) was not understood to be informal support as it did not include an explicit intention to provide such support and similarly, there was a support group of survivors in Frohmann (2005) but there was no explicit aim to improve peer support. Some studies of online platforms, such as blogs (Watanabe et al. 2022) or apps (Ragavan 2019), were excluded because they were not considered to provide informal support per se.

Studies without empirical data or a clear method were excluded. This meant that study protocols were excluded (such as Taft 2009) as were reports without data related to the informal support intervention (such as Mannell 2018).

A group of studies were focused on informal support but did not examine an intervention. These included research by Marin (2012) into the views of victim‐survivors and their supporters but without the presence of an intervention. Similarly, Taylor et al. (2019) focused on the views of DVA helpline workers (some of whom were volunteers) but there was no informal support intervention aimed at this group and helplines were not considered to constitute an informal support intervention.

Studies were excluded because they included mixed populations in the sample, such as Cline (2022) and Edwards et al. (2021), which included survivors of different types of abuse, not just DVA. The sample in Cline (2022) included victim‐survivors of domestic abuse or parental abuse, and Edwards et al. (2021) included victim‐survivors of sexual violence or DVA. Other studies include practitioner samples, such as Goodman (2015), or sampled from a mixed sample of practitioners and volunteers such as the helpline workers in Gregory et al. (2019).

### 5.2 Synthesis of Included Studies

The EGM identified 47 studies examining informal support interventions that ranged from telephone apps for friends to writing groups for victim‐survivors. The overwhelming majority of the interventions/studies were delivered in North America (*n* = 31), mainly in the United States (*n* = 28). Relatively small numbers of interventions were set in Europe (*n* = 5), Asia (*n* = 5), Africa (*n* = 2) and Australasia (*n* = 2). One intervention was online and one was ‘global’ in reach. The characteristics of the included studies are provided in the Summary of Findings in Table 3.

**Table 3:** Summary of findings.

Study author (Year)	Study	Informal support intervention	Outcomes
Abramsky (2014)	**Which country was study/ intervention set in?** Uganda **What was the study design?** 5. Mixed Methods Economics/ cost‐effectiveness **Study Population** Victim‐Survivors Communities **Victim‐Survivor Exposure to DVA (at time of study)** No longer in abusive relationship (Long term) **Victim‐Survivor gender** Not reported **Victim‐Survivor ethnicity/ race** Not reported **Victim‐Survivor age** Adults	**Type of intervention** d) Targeting the community in which ISS takes place **What was the informal social support intervention?** Education/ training Community activities Other (specify)	**Main outcomes/ study tells us about…** Victim‐Survivors Community level Cost estimates **Victim‐Survivor outcomes/ data** Cognitive (knowledge or attitudes) Behavioural Violence or abuse Characteristics of relationship between victim‐survivor and perpetrator Parenting outcomes **Community level outcomes/ data** Awareness and understanding of DVA Social acceptance of DVA Provision of informal social (community) support/ taking action Attitudes towards gender roles Intimate relationships (experiences, views)
Alangea et al. (2020)	**Which country was study/ intervention set in?** Ghana **What was the study design?** 2. Quantitative Randomised Control Trial **Study Population** Victim‐Survivors Informal Supporters Communities **What type of informal supporter?** Community member **Victim‐Survivor Exposure to DVA (at time of study)** Not reported **Victim‐Survivor gender** Not reported **Victim‐Survivor ethnicity/ race** Not reported **Victim‐Survivor age** Adults	**Type of intervention** d) Targeting the community in which ISS takes place **What was the informal social support intervention?** Education/ training Community activities Advocacy (with explicit ISS element)	**Main outcomes/ study tells us about…** Victim‐Survivors Community level **Victim‐Survivor outcomes/ data** Cognitive (knowledge or attitudes) Violence or abuse Characteristics of relationship between victim‐survivor and perpetrator Mental health outcomes **Community level outcomes/ data** Awareness and understanding of DVA Attitudes towards gender roles
Alhusen (2015)	**Which country was study/ intervention set in?** USA **What was the study design?** 1. Qualitative **Study Population** Informal Supporters **What type of informal supporter?** Friend	**Type of intervention** b) Targeting how the support is provided **What was the informal social support intervention?** Education/ training Other (specify)	**Main outcomes/ study tells us about…** Informal Social Supporters **Informal Social Supporter outcomes/ data** Cognitive (knowledge or attitudes) Other (please specify)
Allen (2010)	**Which country was study/ intervention set in?** USA **What was the study design?** 5. Mixed Methods **Study Population** Victim‐Survivors **Victim‐Survivor Exposure to DVA (at time of study)** No longer in abusive relationship (short term) No longer in abusive relationship (Long term) **Victim‐Survivor gender** Female **Victim‐Survivor ethnicity/ race** Ethnic/ racial majority **Victim‐Survivor age** Adults	**Type of intervention** c) Targeting victim‐survivors ability to engage with informal support **What was the informal social support intervention?** Support group	**Main outcomes/ study tells us about…** Victim‐Survivors **Victim‐Survivor outcomes/ data** Social network Psychosocial outcomes Mental health outcomes Housing outcomes
Barr (2018)	**Which country was study/ intervention set in?** USA **What was the study design?** 3. Quantitative non randomised design **Study Population** Victim‐Survivors **Victim‐Survivor Exposure to DVA (at time of study)** No longer in abusive relationship (short term) **Victim‐Survivor gender** Female **Victim‐Survivor ethnicity/ race** Not reported **Victim‐Survivor age** Adults	**Type of intervention** c) Targeting victim‐survivors ability to engage with informal support **What was the informal social support intervention?** Support group	**Main outcomes/ study tells us about…** Victim‐Survivors **Victim‐Survivor outcomes/ data** Social network Psychosocial outcomes
Berg, (2015)	**Which country was study/ intervention set in?** Unspecified (online) **What was the study design?** 5. Mixed Methods **Study Population** Victim‐Survivors **Victim‐Survivor Exposure to DVA (at time of study)** In abusive relationship No longer in abusive relationship (short term) **Victim‐Survivor gender** Female **Victim‐Survivor ethnicity/ race** Ethnic/ racial majority **Victim‐Survivor age** Adults	**Type of intervention** c) Targeting victim‐survivors ability to engage with informal support **What was the informal social support intervention?** Support group	**Main outcomes/ study tells us about…** Victim‐Survivors **Victim‐Survivor outcomes/ data** Cognitive (knowledge or attitudes) Behavioural Social network Psychosocial outcomes
Betron (2021)	**Which country was study/ intervention set in?** Nepal **What was the study design?** 5. Mixed Methods **Study Population** Victim‐Survivors Informal Supporters **What type of informal supporter?** Community member **Victim‐Survivor Exposure to DVA (at time of study)** Not reported **Victim‐Survivor gender** Female **Victim‐Survivor ethnicity/ race** Not reported **Victim‐Survivor age** Adults	**Type of intervention** a) Targeting the provider of informal social support **What was the informal social support intervention?** Education/ training	**Main outcomes/ study tells us about…** Victim‐Survivors Informal Social Supporters Community level **Victim‐Survivor outcomes/ data** Behavioural **Informal Social Supporter outcomes/ data** Cognitive (knowledge or attitudes) Behavioural Other (please specify) **Community level outcomes/ data** Awareness and understanding of DVA Social acceptance of DVA Attitudes towards gender roles Intimate relationships (experiences, views)
Chatterjee (2019)	**Which country was study/ intervention set in?** USA **What was the study design?** 5. Mixed Methods **Study Population** Victim‐Survivors Informal Supporters Practitioners **What type of informal supporter?** Community member **Victim‐Survivor Exposure to DVA (at time of study)** In abusive relationship **Victim‐Survivor gender** Not reported **Victim‐Survivor ethnicity/ race** Ethnic/ racial minority Ethnic/ racial majority **Victim‐Survivor age** Not reported	**Type of intervention** d) Targeting the community in which ISS takes place **What was the informal social support intervention?** Education/ training Community activities	**Main outcomes/ study tells us about…** Victim‐Survivors Community level **Victim‐Survivor outcomes/ data** Behavioural **Community level outcomes/ data** Awareness and understanding of DVA Provision of informal social (community) support/ taking action
Choi et al. (2018)	**Which country was study/ intervention set in?** USA **What was the study design?** 2. Quantitative Randomised Control Trial **Study Population** Informal Supporters **What type of informal supporter?** Faith leader	**Type of intervention** a) Targeting the provider of informal social support **What was the informal social support intervention?** Education/ training	**Main outcomes/ study tells us about…** Informal Social Supporters **Informal Social Supporter outcomes/ data** Cognitive (knowledge or attitudes) Psychosocial outcomes
Choi et al (2023)	**Which country was study/ intervention set in?** USA **What was the study design?** 2. Quantitative Randomised Control Trial **Study Population** Informal Supporters **What type of informal supporter?** Faith leader	**Type of intervention** a) Targeting the provider of informal social support **What was the informal social support intervention?** Education/ training	**Main outcomes/ study tells us about…** Informal Social Supporters **Informal Social Supporter outcomes/ data** Cognitive (knowledge or attitudes) Behavioural
Chu (2020)	**Which country was study/ intervention set in?** China **What was the study design?** 4. Quantitative descriptive **Study Population** Victim‐Survivors **Victim‐Survivor Exposure to DVA (at time of study)** Not reported **Victim‐Survivor gender** Not reported **Victim‐Survivor ethnicity/ race** Not reported **Victim‐Survivor age** Not reported	**Type of intervention** c) Targeting victim‐survivors ability to engage with informal support **What was the informal social support intervention?** Support group	**Main outcomes/ study tells us about…** Victim‐Survivors **Victim‐Survivor outcomes/ data** Behavioural
Coker (1999)	**Which country was study/ intervention set in?** USA **What was the study design?** 1. Qualitative **Study Population** Victim‐Survivors Informal Supporters Practitioners **What type of informal supporter?** Not reported **Victim‐Survivor Exposure to DVA (at time of study)** Not reported **Victim‐Survivor gender** Female **Victim‐Survivor ethnicity/ race** Ethnic/ racial minority **Victim‐Survivor age** Adults	**Type of intervention** b) Targeting how the support is provided **What was the informal social support intervention?** Informal adjudication methods	**Main outcomes/ study tells us about…** Victim‐Survivors
Connolly (2005)	**Which country was study/ intervention set in?** USA **What was the study design?** 1. Qualitative **Study Population** Victim‐Survivors **Victim‐Survivor Exposure to DVA (at time of study)** No longer in abusive relationship (short term) No longer in abusive relationship (Long term) **Victim‐Survivor gender** Female **Victim‐Survivor ethnicity/ race** Ethnic/ racial majority **Victim‐Survivor age** Adults	**Type of intervention** c) Targeting victim‐survivors ability to engage with informal support **What was the informal social support intervention?** Support group	**Main outcomes/ study tells us about…** Victim‐Survivors **Victim‐Survivor outcomes/ data** Cognitive (knowledge or attitudes) Behavioural Psychosocial outcomes Mental health outcomes
Constantino (2005)	**Which country was study/ intervention set in?** USA **What was the study design?** 2. Quantitative Randomised Control Trial **Study Population** Victim‐Survivors **Victim‐Survivor Exposure to DVA (at time of study)** No longer in abusive relationship (short term) **Victim‐Survivor gender** Female **Victim‐Survivor ethnicity/ race** Ethnic/ racial minority Ethnic/ racial majority **Victim‐Survivor age** Adults	**Type of intervention** c) Targeting victim‐survivors ability to engage with informal support **What was the informal social support intervention?** Support group	**Main outcomes/ study tells us about…** Victim‐Survivors **Victim‐Survivor outcomes/ data** Behavioural Social network Psychosocial outcomes Physical health outcomes
Debbonaire et al. (2011)	**Which country was study/ intervention set in?** UK **What was the study design?** 1. Qualitative 3. Quantitative non randomised design **Study Population** Informal Supporters **What type of informal supporter?** Colleague	**Type of intervention** a) Targeting the provider of informal social support **What was the informal social support intervention?** Education/ training Policy development	**Main outcomes/ study tells us about…** Informal Social Supporters **Informal Social Supporter outcomes/ data** Cognitive (knowledge or attitudes) Behavioural
Drumm (2018)	**Which country was study/ intervention set in?** USA **What was the study design?** 3. Quantitative non randomised design **Study Population** Informal Supporters **What type of informal supporter?** Faith leader	**Type of intervention** a) Targeting the provider of informal social support **What was the informal social support intervention?** Education/ training	**Main outcomes/ study tells us about…** Informal Social Supporters **Informal Social Supporter outcomes/ data** Cognitive (knowledge or attitudes) Behavioural Other (please specify)
Edwards (2020)	**Which country was study/ intervention set in?** USA **What was the study design?** 2. Quantitative Randomised Control Trial **Study Population** Informal Supporters **What type of informal supporter?** Peer Friend Family member Community member	**Type of intervention** a) Targeting the provider of informal social support **What was the informal social support intervention?** Education/ training	**Main outcomes/ study tells us about…** Informal Social Supporters **Informal Social Supporter outcomes/ data** Cognitive (knowledge or attitudes) Behavioural Psychosocial outcomes Other (please specify)
Flanigan, (2011)	**Which country was study/ intervention set in?** Canada **What was the study design?** 1. Qualitative 3. Quantitative non randomised design 4. Quantitative descriptive **Study Population** Informal Supporters Communities **What type of informal supporter?** Colleague Community member	**Type of intervention** d) Targeting the community in which ISS takes place **What was the informal social support intervention?** Community activities	**Main outcomes/ study tells us about…** Community level **Community level outcomes/ data** Awareness and understanding of DVA Provision of informal social (community) support/ taking action
Gaarder (2015)	**Which country was study/ intervention set in?** USA **What was the study design?** 1. Qualitative **Study Population** Victim‐Survivors Informal Supporters **What type of informal supporter?** Family member Community member **Victim‐Survivor Exposure to DVA (at time of study)** In abusive relationship **Victim‐Survivor gender** Female **Victim‐Survivor ethnicity/ race** Ethnic/ racial minority Ethnic/ racial majority **Victim‐Survivor age** Adults	**Type of intervention** b) Targeting how the support is provided **What was the informal social support intervention?** Education/ training Informal adjudication methods	**Main outcomes/ study tells us about…** Victim‐Survivors Informal Social Supporters **Victim‐Survivor outcomes/ data** Cognitive (knowledge or attitudes) Behavioural Social network Violence or abuse Characteristics of relationship between victim‐survivor and perpetrator Mental health outcomes Employment or education outcomes **Informal Social Supporter outcomes/ data** Cognitive (knowledge or attitudes) Behavioural Psychosocial outcomes
Glass (2010)	**Which country was study/ intervention set in?** USA **What was the study design?** 5. Mixed Methods **Study Population** Informal Supporters **What type of informal supporter?** Colleague	**Type of intervention** a) Targeting the provider of informal social support **What was the informal social support intervention?** Education/ training	**Main outcomes/ study tells us about…** Informal Social Supporters **Informal Social Supporter outcomes/ data** Cognitive (knowledge or attitudes)
Gordon (1996)	**Which country was study/ intervention set in?** USA **What was the study design?** 5. Mixed Methods **Study Population** Victim‐Survivors **Victim‐Survivor Exposure to DVA (at time of study)** In abusive relationship No longer in abusive relationship (short term) No longer in abusive relationship (Long term) **Victim‐Survivor gender** Female **Victim‐Survivor ethnicity/ race** Ethnic/ racial minority Ethnic/ racial majority **Victim‐Survivor age** Adults	**Type of intervention** c) Targeting victim‐survivors ability to engage with informal support **What was the informal social support intervention?** Support group	**Main outcomes/ study tells us about…** Victim‐Survivors **Victim‐Survivor outcomes/ data** Social network Psychosocial outcomes
Graham‐Bermann and Miller (2013)	**Which country was study/ intervention set in?** USA **What was the study design?** 2. Quantitative Randomised Control Trial **Study Population** Victim‐Survivors **Victim‐Survivor Exposure to DVA (at time of study)** In abusive relationship No longer in abusive relationship (short term) **Victim‐Survivor gender** Female **Victim‐Survivor ethnicity/ race** Ethnic/ racial minority Ethnic/ racial majority **Victim‐Survivor age** Adults	**Type of intervention** c) Targeting victim‐survivors ability to engage with informal support **What was the informal social support intervention?** Support group	**Main outcomes/ study tells us about…** Victim‐Survivors **Victim‐Survivor outcomes/ data** Mental health outcomes
Hancock (2014)	**Which country was study/ intervention set in?** USA **What was the study design?** 4. Quantitative descriptive **Study Population** Informal Supporters **What type of informal supporter?** Faith leader	**Type of intervention** a) Targeting the provider of informal social support **What was the informal social support intervention?** Education/ training	**Main outcomes/ study tells us about…** Informal Social Supporters **Informal Social Supporter outcomes/ data** Cognitive (knowledge or attitudes)
Kim (2011)	**Which country was study/ intervention set in?** USA **What was the study design?** 5. Mixed Methods **Study Population** Victim‐Survivors Informal Supporters Practitioners Communities **What type of informal supporter?** Community member **Victim‐Survivor Exposure to DVA (at time of study)** No longer in abusive relationship (short term) No longer in abusive relationship (Long term) **Victim‐Survivor gender** Female **Victim‐Survivor ethnicity/ race** Ethnic/ racial minority Ethnic/ racial majority **Victim‐Survivor age** Adults	**Type of intervention** c) Targeting victim‐survivors ability to engage with informal support **What was the informal social support intervention?** Support group	**Main outcomes/ study tells us about…** Victim‐Survivors Community level **Victim‐Survivor outcomes/ data** Cognitive (knowledge or attitudes) Behavioural Social network Psychosocial outcomes Mental health outcomes **Community level outcomes/ data** Awareness and understanding of DVA Confidence in providing ISS
Kowanko and Power (2008)	**Which country was study/ intervention set in?** Australia **What was the study design?** 1. Qualitative **Study Population** Informal Supporters Communities **What type of informal supporter?** Community member	**Type of intervention** d) Targeting the community in which ISS takes place **What was the informal social support intervention?** Community activities Support group	**Main outcomes/ study tells us about…** Community level **Community level outcomes/ data** Provision of informal social (community) support/ taking action
Lindgren (2014)	**Which country was study/ intervention set in?** Sweden **What was the study design?** 4. Quantitative descriptive **Study Population** Victim‐Survivors **Victim‐Survivor Exposure to DVA (at time of study)** Not reported **Victim‐Survivor gender** Not reported **Victim‐Survivor ethnicity/ race** Not reported **Victim‐Survivor age** Not reported	**Type of intervention** c) Targeting victim‐survivors ability to engage with informal support **What was the informal social support intervention?** Support group	**Main outcomes/ study tells us about…** Victim‐Survivors **Victim‐Survivor outcomes/ data** Behavioural Social network Psychosocial outcomes Mental health outcomes
Macy (2012)	**Which country was study/ intervention set in?** USA **What was the study design?** 1. Qualitative **Study Population** Victim‐Survivors Practitioners **Victim‐Survivor Exposure to DVA (at time of study)** Not reported **Victim‐Survivor gender** Female **Victim‐Survivor ethnicity/ race** Ethnic/ racial minority Ethnic/ racial majority **Victim‐Survivor age** Adults	**Type of intervention** c) Targeting victim‐survivors ability to engage with informal support **What was the informal social support intervention?** Support group	**Main outcomes/ study tells us about…** Victim‐Survivors **Victim‐Survivor outcomes/ data** Cognitive (knowledge or attitudes) Behavioural Social network Violence or abuse Characteristics of relationship between victim‐survivor and perpetrator Economic outcomes Parenting outcomes
Magnussen (2019)	**Which country was study/ intervention set in?** USA **What was the study design?** 3. Quantitative non randomised design **Study Population** Informal Supporters Communities **What type of informal supporter?** Community member	**Type of intervention** d) Targeting the community in which ISS takes place **What was the informal social support intervention?** Community activities	**Main outcomes/ study tells us about…** Community level **Community level outcomes/ data** Awareness and understanding of DVA Social acceptance of DVA Provision of informal social (community) support/ taking action Confidence in providing ISS
Mahapatro and Singh (2020)	**Which country was study/ intervention set in?** India **What was the study design?** 5. Mixed Methods **Study Population** Victim‐Survivors **Victim‐Survivor Exposure to DVA (at time of study)** In abusive relationship **Victim‐Survivor gender** Female **Victim‐Survivor ethnicity/ race** Not reported **Victim‐Survivor age** Adults	**Type of intervention** c) Targeting victim‐survivors ability to engage with informal support **What was the informal social support intervention?** Other (specify) Counselling/therapy (with explicit ISS element)	**Main outcomes/ study tells us about…** Victim‐Survivors **Victim‐Survivor outcomes/ data** Behavioural Violence or abuse Characteristics of relationship between victim‐survivor and perpetrator Psychosocial outcomes Mental health outcomes Economic outcomes
Molina (2009)	**Which country was study/ intervention set in?** USA **What was the study design?** 1. Qualitative **Study Population** Victim‐Survivors **Victim‐Survivor Exposure to DVA (at time of study)** No longer in abusive relationship (short term) No longer in abusive relationship (Long term) **Victim‐Survivor gender** Female **Victim‐Survivor ethnicity/ race** Ethnic/ racial minority **Victim‐Survivor age** Adults	**Type of intervention** c) Targeting victim‐survivors ability to engage with informal support **What was the informal social support intervention?** Support group	**Main outcomes/ study tells us about…** Victim‐Survivors **Victim‐Survivor outcomes/ data** Cognitive (knowledge or attitudes) Behavioural Social network Psychosocial outcomes Parenting outcomes
Morales‐Campos (2009)	**Which country was study/ intervention set in?** USA **What was the study design?** 1. Qualitative **Study Population** Victim‐Survivors **Victim‐Survivor Exposure to DVA (at time of study)** In abusive relationship **Victim‐Survivor gender** Female **Victim‐Survivor ethnicity/ race** Ethnic/ racial minority **Victim‐Survivor age** Adults	**Type of intervention** c) Targeting victim‐survivors ability to engage with informal support **What was the informal social support intervention?** Support group	**Main outcomes/ study tells us about…** Victim‐Survivors **Victim‐Survivor outcomes/ data** Social network Psychosocial outcomes
Pillinger (2020)	**Which country was study/ intervention set in?** global **What was the study design?** 1. Qualitative 4. Quantitative descriptive **Study Population** Informal Supporters **What type of informal supporter?** Colleague	**Type of intervention** a) Targeting the provider of informal social support **What was the informal social support intervention?** Education/ training Policy development	**Main outcomes/ study tells us about…** Informal Social Supporters **Informal Social Supporter outcomes/ data** Cognitive (knowledge or attitudes) Behavioural
Prosman (2014)	**Which country was study/ intervention set in?** Netherlands **What was the study design?** 3. Quantitative non randomised design **Study Population** Victim‐Survivors **Victim‐Survivor Exposure to DVA (at time of study)** In abusive relationship **Victim‐Survivor gender** Female **Victim‐Survivor ethnicity/ race** Ethnic/ racial minority Ethnic/ racial majority **Victim‐Survivor age** Adults	**Type of intervention** a) Targeting the provider of informal social support **What was the informal social support intervention?** Education/ training Advocacy (with explicit ISS element) Mentoring/ befriending	**Main outcomes/ study tells us about…** Victim‐Survivors **Victim‐Survivor outcomes/ data** Behavioural Social network Violence or abuse Mental health outcomes Employment or education outcomes
Ross (2013)	**Which country was study/ intervention set in?** USA **What was the study design?** 5. Mixed Methods **Study Population** Victim‐Survivors **Victim‐Survivor Exposure to DVA (at time of study)** No longer in abusive relationship (short term) **Victim‐Survivor gender** Female **Victim‐Survivor ethnicity/ race** Ethnic/ racial minority Ethnic/ racial majority **Victim‐Survivor age** Adults	**Type of intervention** c) Targeting victim‐survivors ability to engage with informal support **What was the informal social support intervention?** Education/ training Support group	**Main outcomes/ study tells us about…** Victim‐Survivors **Victim‐Survivor outcomes/ data** Cognitive (knowledge or attitudes) Behavioural Psychosocial outcomes
Santos et al. (2017)	**Which country was study/ intervention set in?** Portugal **What was the study design?** 3. Quantitative non randomised design **Study Population** Victim‐Survivors **Victim‐Survivor Exposure to DVA (at time of study)** In abusive relationship No longer in abusive relationship (short term) **Victim‐Survivor gender** Female **Victim‐Survivor ethnicity/ race** Ethnic/ racial minority Ethnic/ racial majority **Victim‐Survivor age** Adults	**Type of intervention** c) Targeting victim‐survivors ability to engage with informal support **What was the informal social support intervention?** Support group	**Main outcomes/ study tells us about…** Victim‐Survivors **Victim‐Survivor outcomes/ data** Psychosocial outcomes Mental health outcomes
Schuler (2011)	**Which country was study/ intervention set in?** Vietnam **What was the study design?** 1. Qualitative **Study Population** Victim‐Survivors Informal Supporters **What type of informal supporter?** neighbour **Victim‐Survivor Exposure to DVA (at time of study)** Not reported **Victim‐Survivor gender** Female **Victim‐Survivor ethnicity/ race** Not reported **Victim‐Survivor age** Adults	**Type of intervention** d) Targeting the community in which ISS takes place **What was the informal social support intervention?** Education/ training Community activities Advocacy (with explicit ISS element)	**Main outcomes/ study tells us about…** Victim‐Survivors Community level **Victim‐Survivor outcomes/ data** Cognitive (knowledge or attitudes) Behavioural Social network Psychosocial outcomes **Community level outcomes/ data** Awareness and understanding of DVA Social acceptance of DVA Provision of informal social (community) support/ taking action
Sullivan (1999)	**Which country was study/ intervention set in?** USA **What was the study design?** 1. Qualitative 2. Quantitative Randomised Control Trial **Study Population** Victim‐Survivors **Victim‐Survivor Exposure to DVA (at time of study)** No longer in abusive relationship (short term) **Victim‐Survivor gender** Female **Victim‐Survivor ethnicity/ race** Ethnic/ racial minority Ethnic/ racial majority **Victim‐Survivor age** Adults	**Type of intervention** c) Targeting victim‐survivors ability to engage with informal support **What was the informal social support intervention?** Advocacy (with explicit ISS element)	**Main outcomes/ study tells us about…** Victim‐Survivors **Victim‐Survivor outcomes/ data** Behavioural Social network Violence or abuse Psychosocial outcomes Mental health outcomes Physical health outcomes Employment or education outcomes Economic outcomes Parenting outcomes Housing outcomes
Taft (2011)	**Which country was study/ intervention set in?** Australia **What was the study design?** 2. Quantitative Randomised Control Trial **Study Population** Victim‐Survivors **Victim‐Survivor Exposure to DVA (at time of study)** In abusive relationship **Victim‐Survivor gender** Female **Victim‐Survivor ethnicity/ race** Ethnic/ racial minority Ethnic/ racial majority **Victim‐Survivor age** Adults	**Type of intervention** a) Targeting the provider of informal social support **What was the informal social support intervention?** Education/ training Advocacy (with explicit ISS element) Other (specify) Mentoring/ befriending	**Main outcomes/ study tells us about…** Victim‐Survivors **Victim‐Survivor outcomes/ data** Behavioural Social network Violence or abuse Psychosocial outcomes Mental health outcomes Physical health outcomes Parenting outcomes
Taha (2015)	**Which country was study/ intervention set in?** USA **What was the study design?** 2. Quantitative Randomised Control Trial **Study Population** Victim‐Survivors **Victim‐Survivor Exposure to DVA (at time of study)** In abusive relationship No longer in abusive relationship (short term) **Victim‐Survivor gender** Female **Victim‐Survivor ethnicity/ race** Ethnic/ racial minority **Victim‐Survivor age** Adults	**Type of intervention** c) Targeting victim‐survivors ability to engage with informal support **What was the informal social support intervention?** Support group	**Main outcomes/ study tells us about…** Victim‐Survivors **Victim‐Survivor outcomes/ data** Behavioural Psychosocial outcomes Mental health outcomes
Tam (2004)	**Which country was study/ intervention set in?** Canada **What was the study design?** 1. Qualitative **Study Population** Victim‐Survivors Practitioners **Victim‐Survivor Exposure to DVA (at time of study)** In abusive relationship No longer in abusive relationship (short term) **Victim‐Survivor gender** Female **Victim‐Survivor ethnicity/ race** Ethnic/ racial minority **Victim‐Survivor age** Adults	**Type of intervention** c) Targeting victim‐survivors ability to engage with informal support **What was the informal social support intervention?** Advocacy (with explicit ISS element)	**Main outcomes/ study tells us about…** Victim‐Survivors **Victim‐Survivor outcomes/ data** Cognitive (knowledge or attitudes) Behavioural Psychosocial outcomes Economic outcomes Housing outcomes Culturally specific outcomes/ needs
Tan et al. (1995)	**Which country was study/ intervention set in?** USA **What was the study design?** 2. Quantitative Randomised Control Trial **Study Population** Victim‐Survivors **Victim‐Survivor Exposure to DVA (at time of study)** In abusive relationship No longer in abusive relationship (short term) **Victim‐Survivor gender** Female **Victim‐Survivor ethnicity/ race** Ethnic/ racial minority Ethnic/ racial majority **Victim‐Survivor age** Adults	**Type of intervention** c) Targeting victim‐survivors ability to engage with informal support **What was the informal social support intervention?** Advocacy (with explicit ISS element)	**Main outcomes/ study tells us about…** Victim‐Survivors **Victim‐Survivor outcomes/ data** Behavioural Social network Violence or abuse Psychosocial outcomes Mental health outcomes
Taylor (2000)	**Which country was study/ intervention set in?** USA **What was the study design?** 1. Qualitative **Study Population** Victim‐Survivors **Victim‐Survivor Exposure to DVA (at time of study)** No longer in abusive relationship (short term) No longer in abusive relationship (Long term) **Victim‐Survivor gender** Female **Victim‐Survivor ethnicity/ race** Ethnic/ racial minority **Victim‐Survivor age** Adults	**Type of intervention** c) Targeting victim‐survivors ability to engage with informal support **What was the informal social support intervention?** Support group	**Main outcomes/ study tells us about…** Victim‐Survivors **Victim‐Survivor outcomes/ data** Behavioural Social network Psychosocial outcomes
Tutty et al. (2016)	**Which country was study/ intervention set in?** Canada **What was the study design?** 1. Qualitative **Study Population** Informal Supporters **What type of informal supporter?** Peer	**Type of intervention** c) Targeting victim‐survivors ability to engage with informal support **What was the informal social support intervention?** Support group	**Main outcomes/ study tells us about…** Informal Social Supporters **Informal Social Supporter outcomes/ data** Cognitive (knowledge or attitudes) Behavioural
Women's Aid (2020)	**Which country was study/ intervention set in?** UK **What was the study design?** 5. Mixed Methods **Study Population** Informal Supporters **What type of informal supporter?** Community member	**Type of intervention** d) Targeting the community in which ISS takes place **What was the informal social support intervention?** Education/ training Community activities	**Main outcomes/ study tells us about…** Informal Social Supporters **Informal Social Supporter outcomes/ data** Cognitive (knowledge or attitudes) Behavioural Other (please specify)
Wong et al. (2019)	**Which country was study/ intervention set in?** China **What was the study design?** 5. Mixed Methods **Study Population** Informal Supporters **What type of informal supporter?** Peer Friend	**Type of intervention** a) Targeting the provider of informal social support **What was the informal social support intervention?** Education/ training	**Main outcomes/ study tells us about…** Informal Social Supporters **Informal Social Supporter outcomes/ data** Cognitive (knowledge or attitudes) Behavioural
Zakheim (2009)	**Which country was study/ intervention set in?** USA **What was the study design?** 1. Qualitative **Study Population** Victim‐Survivors Practitioners **Victim‐Survivor Exposure to DVA (at time of study)** In abusive relationship **Victim‐Survivor gender** Female Male **Victim‐Survivor ethnicity/ race** Ethnic/ racial majority **Victim‐Survivor age** Adults	**Type of intervention** b) Targeting how the support is provided **What was the informal social support intervention?** Informal adjudication methods	**Main outcomes/ study tells us about…** Victim‐Survivors **Victim‐Survivor outcomes/ data** Cognitive (knowledge or attitudes) Social network Violence or abuse Psychosocial outcomes
Zlotnick (2011)	**Which country was study/ intervention set in?** USA **What was the study design?** 2. Quantitative Randomised Control Trial **Study Population** Victim‐Survivors **Victim‐Survivor Exposure to DVA (at time of study)** In abusive relationship **Victim‐Survivor gender** Female **Victim‐Survivor ethnicity/ race** Ethnic/ racial minority Ethnic/ racial majority **Victim‐Survivor age** Adults	**Type of intervention** c) Targeting victim‐survivors ability to engage with informal support **What was the informal social support intervention?** Counselling/therapy (with explicit ISS element)	**Main outcomes/ study tells us about…** Victim‐Survivors **Victim‐Survivor outcomes/ data** Violence or abuse Mental health outcomes

Grey literature was an important part of the EGM with 16 included studies reported in non‐commercial publications, with the majority constituting PhD theses and third sector reports. Handsearching and other methods also played an important role in the EGM, identifying 16 studies, 12 of which were found through citation chasing of references from relevant systematic reviews and included studies.

#### 5.2.1 Informal Social Support Interventions

The EGM included studies of a diverse set of interventions, distributed across all four types of ISS interventions outlined in the framework above (see Figure 1). The majority of interventions were tailored towards type c) victim‐survivors' capacity to engage with and utilise informal support (*n* = 23). Fewer interventions targeted type (a) the provider of informal support (*n* = 12), and type (d) community‐wide interventions (*n* = 8). A smaller group of interventions primarily focused on type (b) the nature and quality of the relationship between informal supporter and victim‐survivor (*n* = 4). Further details about the findings for each of these intervention types are outlined below.

a. *ISS interventions targeting the provider of informal support* (*n* = 12).
  This set of interventions targeted individual‐level and personal factors that influence how informal supporters behave in response to disclosures of abuse/victim‐survivors. Interventions included educational activities for specific informal supporters and befriending/mentoring activities that aimed to mobilise support from specific individuals who may be predisposed to providing positive support, by virtue of their personal situation/history.
  Interventions targeting the informal supporter primarily included *educational activities* designed to develop knowledge and skills to effectively respond to victim‐survivors. Interventions were tailored towards existing and potential informal supporters in various settings: Faith Leaders in places of worship (*n* = 4), workplace supervisors and colleagues (*n* = 3), student peers in colleges (*n* = 2) and volunteers from the wider community (*n* = 3).
  The nature of the educational activities varied but mainly included one‐off training sessions of varying durations. In‐person training included presentations in the workplace and staff briefing sessions (Debbonaire et al. 2011) and a 4‐h workshop for Faith Leaders (Drumm 2018). Online training included courses ranging from 75 min (Glass 2010) to 3 h (Hancock 2014), and simulation training (Choi 2023). One intervention also included a booster session after an initial 2‐h workshop (Edwards et al. 2020). Alongside training, two interventions included the development of DVA policies in the workplace.
  A subset of interventions focused on creating non‐professional mentor mothers who would directly provide informal support by ‘establishing a friendly supportive relationship with the abused women’ (Prosman 2014). Such interventions included longer training programmes for volunteers, between 5 to 10 days of training, and covered a broad curriculum (Taft 2011; Prosman 2014). Such *befriending interventions* were premised on the mothering role that both victim‐survivor and informal supporter held. Therefore, the interventions also included additional elements such as advocacy and parenting support.
b. *ISS interventions that shape the quality of support and/or relationship between informal supporters and victim‐survivors* (*n* = 4).
  This set of interventions focused on interpersonal relationships, harnessing existing positive channels of support and shaping the interactions between victim‐survivors and their informal networks through mediation methods and technological aids.
  Four interventions focused on improving the quality and nature of responses from existing informal supporters. Three of the interventions included *informal adjudication approaches* that brought victim‐survivors together with their informal supporters through ‘support’ or ‘healing’ circles (Gaarder 2015; Zakheim 2009) or ‘peacemaking’ sessions (Coker 1999). Such initiatives tended to involve family members and key members of the community coming together with the victim‐survivor to discuss their situation, and offer support and resolutions. Two of the interventions were rooted in cultural traditions: Jewish (Zakheim 2009) and Navajo (Coker 1999).
  One intervention included a *smartphone application* aimed at both victim‐survivors and their female friends (Alhusen et al. 2015). This application functioned as an interactive, personalised decision aid that also provided guidance for female informal supporters on ‘how to best support their friends or family members while keeping safe’.
c. *ISS interventions that primarily focus on victim‐survivors' ability to engage with, and utilise, informal support* (*n* = 23).
  This set of interventions was tailored towards the personal situation of the victim‐survivor and individual‐level factors that shape their ability to engage with informal support. Interventions included DVA support groups, advocacy with an explicit intention to foster informal support, and counselling for victim‐survivors with a specific emphasis on informal support relationships.
*Support groups:* The majority of interventions in this category were support groups for victim‐survivors (*n* = 18). These groups enabled victim‐survivors to make connections and gain support from peers who also had lived experience of abuse. Such interventions were ‘designed to provide emotional, psychological, educational and sometimes practical support to groups of individuals who share a problem or situation’ and may have been facilitated ‘by professionals, paraprofessionals or peers (or a combination thereof)’ (Sullivan 2012, 3). The support groups were diverse in format, including traditional discussion groups that provided an environment to chat with friends (Constantino et al. 2005), alongside healing or therapeutic activities, such as ‘meditation, yoga, creative visualisation and art therapy’ (Allen and Wozniak 2010), writing (Barr 2018) and/or art‐making (Kim 2011). The support groups took place in diverse settings and so informal support was provided via groups hosted in non‐governmental organisations (*n* = 5), supported housing (*n* = 2), community settings (*n* = 2) and/or across multiple health and community settings (*n* = 1). Six studies did not indicate where the support groups, and so informal support, was being provided. The EGM also included support groups operating via online forums/platforms (Berg 2015; Chu et al. 2020).
  Four support groups were tailored towards particular cultural backgrounds, with three including migrated populations: Latina immigrant women (Molina et al. 2009), Hispanic women (Morales‐Campos et al. 2009) and African American women (Taha et al. 2015; Taylor 2000). Support groups mainly included women victim‐survivors (*n* = 15) who either remained in abusive relationships (*n* = 6), or were no longer in the abusive relationship in the short (*n* = 13) and/or longer terms (*n* = 6). Three studies did not report the victim‐survivor's relationship status. Where reported, members of the support groups were from both ethnic minority and majority groups (*n* = 10) in the country of study, only ethnic majorities (*n* = 3) or minorities (*n* = 4).
  One study examined support groups as one part of a larger recovery programme (Connolly 2005) and one study included training for peer support alongside their involvement in support groups (Ross 2013).
*Advocates that foster informal support:* Three interventions included advocates working directly with victim‐survivors to help them ‘access the necessary resources, experience empowerment and expand social support’ (Tam 2004). These interventions explicitly focused on ‘making the community more responsive’ (Sullivan and Bybee 1999, 45) and actively engaging with friends or relatives of the victim‐survivor.
*Counselling or therapy to enhance victim‐survivor's engagement with informal support:* Two interventions included counselling or interpersonal psychotherapy that placed ‘emphasis on the enhancement of social support’ and ‘strengthening social relationships’ (Zlotnick et al. 2011).
d. *ISS interventions that target the community within which the informal support takes place* (*n* = 8).
  This set of interventions targeted community‐ or societal‐level factors that can engender or inhibit informal support. The interventions focused on generic community members who did not have pre‐existing support relationships with victim‐survivors.
  All interventions involved *community‐based activities* tailored towards the community at large. The majority of interventions (*n* = 5) included community capacity‐building activities to improve collective understanding and foster social norms that are conducive to positive responses to victim‐survivors of DVA. Interventions trained volunteers from the locality to serve as community‐based activists (e.g., Community Ambassadors, Women's Aid 2020) or teams (e.g., Community‐Based Action Teams, Alangea et al. 2020) to bolster informal support through community channels for victim‐survivors. Collaboration with stakeholders and community members was typically intended to ‘spread awareness about DV and to bring a positive change’ in the community (Chatterjee 2019). Interventions undertook a broad range of public and community activities, including ‘awareness‐raising activities, campaigning and fundraising activities’ (Women's Aid 2020), to drive improvements in community responses to DVA victim‐survivors. Specific examples of community‐based activities included culturally targeted interventions, for example, Hawaian ‘talkstory’ discussion groups which provide informal platforms for ‘reciprocal exchange of thoughts, ideas, feelings about self’ (Magnussen et al. 2019, 170). This intervention aimed to help participants reflect critically on traditional practices and assumptions that shape perceptions of DVA in their communities, and identify community support and safety strategies for victim‐survivors of DVA. The interventions often utilised multiple channels to raise awareness about DVA and mobilise the community into action: ‘community dramas, discussions and meetings; small group activities; one‐on‐one “quick chats”; door‐to‐door discussions; trainings; poster discussions; and film and soap opera shows’ (Abramsky et al. 2014).
  Community‐focused interventions were implemented in a wide range of geographical areas including the United States, United Kingdom, Canada, Australia, Uganda, Ghana and Vietnam. As such, some of these interventions were explicitly tailored to a particular community or cultural setting.

#### 5.2.2 Study Population

The EGM only included studies that reported on victim‐survivors of DVA (*n* = 25), informal supporters (*n* = 15) or both (*n* = 7). Some studies (*n* = 6) also reported data for practitioners/formal services, such as policing and healthcare professionals (Chatterjee 2019) or service providers (Macy et al. 2012; Tam 2004). Outcomes or data for practitioners/formal providers were not analysed as part of the EGM.

A high number of studies included *victim‐survivors* in the sample population (*n* = 32). This reflects the high proportion of interventions tailored towards this group (i.e., 23 studies focused on type c interventions: those that targeted victim‐survivors) as such studies collected data from individuals targeted by the intervention. Other intervention types, whether directly targeting victim‐survivors or not, also reported data for victim‐survivor samples. A few studies (*n* = 7) evaluated the impacts of informal support interventions not only for the direct beneficiaries of the intervention, that is, specific informal supporters (type a) or communities (type d) but also for downstream impacts on individual victim‐survivors. These studies examined the consequences of community activities (Alangea 2020; Abramsky 2014; Chatterjee 2019; Schuler 2011) and mentoring interventions (Betron 2021; Prosman 2014; Taft 2011) for victim‐survivors.

The vast majority of sampled victim‐survivors were female adults. One study also included male victim‐survivors and four studies did not report the gender of the sample. The victim‐survivors were still in the abusive relationship (*n* = 15) and/or had left recently (*n* = 15) and/or over a year ago (*n* = 7). Seven studies did not report victim‐survivors' exposure to DVA at the time of the study. Where reported, victim‐survivor samples included ethnic minority and majority groups in the country of study (*n* = 14), majority (*n* = 4) or minority (*n* = 6) only samples. The latter set of studies focused specifically on ethnic minorities in the North American context, including Navajo women (Coker 1999), Chinese‐Canadian women (Tam 2004), Latina female immigrants (Molina 2009), Hispanic women immigrants (Morales‐Campos 2009) and African American women (Taha 2015; Taylor 2000).

Data was collected from *informal supporters* in 22 studies. These included friends (*n* = 3), family (*n* = 2), colleagues (*n* = 5), neighbours (*n* = 1) and peers (*n* = 4) of victim‐survivors as well as Faith Leaders (*n* = 4) and wider members of the community (*n* = 13). A majority of studies (*n* = 15) included informal supporters who were reported to share similar experiences or backgrounds with the victim‐survivors. Informal supporters in seven studies were reported to share a common language or culture with the victim‐survivors, and eight studies included supporters who had personal experience of relationship abuse.

#### 5.2.3 Study Outcomes

Most studies reported individual‐level outcomes for victim‐survivors (*n* = 32) and/or informal supporters (*n* = 14). Relationship level outcomes, that is, data on the characteristics of relationships between victim‐survivor and perpetrator, or victim‐survivors and their social network, were reported in fewer studies (*n* = 18). Community‐level outcomes, representing data for the whole population/community group were least frequently collected and reported (*n* = 9). For *victim‐survivors*, the highest proportion of studies focused on their psycho‐social and/or mental health (*n* = 26). The second most frequent outcome type for victim‐survivors focused on their behaviour (*n* = 22), and whether they had sought or obtained help and support. Studies focused on whether informal support interventions were associated with seeking help from formal services such as healthcare or criminal justice agencies (*n* = 11), seeking further informal sources of support (*n* = 6) and/or receiving informal support (*n* = 10). Further outcomes included victim‐survivors' ongoing involvement with the abusive partner (*n* = 5), desire to make changes in their relationship (*n* = 2) and sexual behaviours (*n* = 1). The third most common group reported by studies focused on relationship‐level outcomes with a focus on the victim‐survivors' relationship with their social support network, and data on the size and strength of such networks (*n* = 18).

Studies also examined individual cognitive outcome categories, focusing on the knowledge and attitudes of victim‐survivors (*n* = 12). These studies examined victim‐survivors' awareness and understanding of DVA (*n* = 11) as well as attitudes towards gender roles (*n* = 2) and knowledge of sources of support (*n* = 1). A subset of studies focused on victim‐survivors' reports of ongoing relationship violence and abuse (*n* = 11) and the nature of the relationship with the perpetrator (*n* = 5). Further outcomes included parenting (*n* = 5), economics/finances (*n* = 4), housing (*n* = 3), employment or education (*n* = 3), physical health (*n* = 3) and culturally specific needs (*n* = 1).

Studies reporting outcomes or data for *informal supporters* were relatively few (*n* = 14). All these studies reported on cognitive outcome types, specifically informal supporters' awareness and understanding of DVA (*n* = 14) and their confidence in providing support (*n* = 13). Studies also reported on their knowledge of how to respond to disclosures of abuse (*n* = 6) and knowledge about their own emotional well‐being (*n* = 2).

The EGM also included studies that reported behavioural outcomes for informal supporters (*n* = 10), which included providing support (*n* = 10) and reactions to disclosure (*n* = 2). Further, psycho‐social outcomes were reported by three studies.

Only nine studies in the EGM reported *community‐*level outcomes so most studies did not (*n* = 38). Those studies that did (*n* = 9) tended to focus on cognitive outcomes, specifically community awareness and understanding of DVA (*n* = 8), social attitudes towards gender roles (*n* = 2) and acceptability of DVA (*n* = 4), and confidence in providing support (*n* = 2). Six studies reported on behavioural outcomes, specifically on whether communities were, or were perceived to be, actively supporting victim‐survivors (using qualitative and quantitative data sources).

#### 5.2.4 Study Design

The EGM included diverse study designs. The largest set of studies used qualitative designs (*n* = 17). Mixed method approaches, integrating qualitative and quantitative methods, were the second largest set (*n* = 12), followed by Randomised Controlled Trials (*n* = 11). The EGM also included non‐randomised (*n* = 7) and descriptive quantitative (*n* = 5) designs, with one study also undertaking cost‐effectiveness analysis.

The online interactive Evidence and Gap Map is available here: https://eppi.ioe.ac.uk/cms/Portals/35/Maps/DVA_Informal_Support.html.

## 6 Discussion

### 6.1 Summary of Main Results

This evidence and gap map included 47 studies of interventions that aimed to create, enhance or facilitate informal support for victim‐survivors of DVA. The evidence base highlights that such interventions are multiple, varied, and designed and delivered around the world. The evidence base is unevenly distributed with the greatest concentration of studies on interventions targeting victim‐survivors in the Global North and focusing on female populations, with a particular emphasis on mental health and wellbeing outcomes. A large proportion of interventions in the EGM targeted individual‐level factors rather than relational or community dynamics as per the socio‐ecological framework of DVA (Heise 1998; World Health Organisation 2021).

A defining feature of the evidence base captured by the EGM is that victim‐survivors are foregrounded by the studies, whether as a focus for the interventions or the source of study data. Almost half of the informal support interventions (*n* = 23) were tailored towards victim‐survivors' ability to engage with their peers, familial and/or wider social networks (type c interventions). As such, these interventions targeted the individual victim‐survivor and their personal situation, e.g., their emotional and psychological disposition to engage with informal networks. These interventions target social isolation associated with DVA, as wider studies highlight that women experiencing abuse, compared to those who do not, report impaired social support (Levendosky et al. 2004; Katerndahl et al. 2012).

Victim‐survivors constituted the study sample in a high proportion of the studies in the EGM, which reported the perceptions, experiences or outcomes of female adult victim‐survivors (*n* = 32). Foregrounding victim‐survivors is important in the field of DVA and potentially represents the ethical imperative behind much DVA research to improve the lives of victim‐survivors of abuse (Women's Aid 2020). The populations in the studies were almost exclusively female victim‐survivors, rather than from male or non‐binary groups. This speaks to the gendered nature of domestic abuse as evidence points to greater prevalence and impacts amongst women (Cunningham and Anderson 2023; Fanslow et al. 2023) and so may explain the increased targeting of interventions and research towards female groups. Moreover, wider research also suggests that informal support is gendered, with female victim‐survivors valuing informal sources of support and female friends, family and colleagues playing a greater role in providing support compared to male counterparts (Gregory and Williamson 2021; Sylaska and Edwards 2014). Studies also highlight that ethnicity plays an important role in shaping victim‐survivors' preference for informal, rather than formal, channels of support (Fiolet et al. 2019; Ragavan et al. 2018; Rivas et al. 2013; Rizo et al. 2011). Almost half of the studies in the EGM reported the ethnicity of the population group (*n* = 24), with a set of interventions tailored towards specific ethnic minority women in North America.

The mental health and wellbeing of victim‐survivors was the most frequent outcome category reported by studies (*n* = 26), likely driven by the association between social support and improved mental health established by the wider literature (e.g., Beeble et al. 2009; Coker et al. 2002; Sapkota et al. 2022). Similarly, studies have established a link between improved informal support and further help‐seeking (Liang et al. 2005; Shin and Park 2020) and so this may explain the focus on victim‐survivor behaviours as the second largest set of outcomes.

The evidence base captured by the EGM included diverse study designs with over half of the research employing qualitative and mixed method approaches. The inclusion of qualitative DVA research is understood to make unique contributions to our assessment of interventions (Powell et al. 2023) but a lack of robust experimental study designs is also considered to inhibit our knowledge of effectiveness (Feder et al. 2011; Trabold et al. 2018). Non‐academic research, which tends towards qualitative or descriptive quantitative approaches, played an important part in this EGM and reflects the wider utility of grey sources in the field of DVA (Casey et al. 2020) and specifically informal support research (e.g., Konya et al. 2020; Ogbe et al. 2020).

### 6.2 Areas of Major Gaps in the Evidence

This EGM identifies several major gaps in the research: (1) Informal support interventions per se, and interventions specifically targeting the relationship between victim‐survivor and informal supporter (type b) and communities (type d), (2) Specific types of victim‐survivor population including those that have left the abusive relationship in the longer term, and friends or relatives of victim‐survivors, (3) Studies designed to rigorously assess the impacts of the intervention and (4) Outcomes.

1. *Informal social support interventions*
  Whilst the EGM identified multiple informal support interventions, it is clear that there are many more initiatives, and of greater diversity, being implemented around the world. Therefore, this EGM provides a partial picture of interventions aiming to create or enhance informal support for victim‐survivors of DVA. In the United Kingdom, for example, only two interventions were included in the EGM but the authors are aware that other programmes are being designed and delivered. These include, for example, the Findaway project in Sunderland, UK (https://www.wefindaway.org.uk/) and the Bright Sky telephone App/website (https://www.hestia.org/brightsky). Such interventions are likely not included in the EGM because they have not (yet) been subject to research and/or such research is not publicly available. Therefore, as other EGMs have noted, the gaps are in the research evidence rather than the lack of interventions (Sydes et al. 2023). Several factors may explain this. First, policy paradigms and priorities shape DVA interventions and associated evaluation research (e.g., Davies 2018; Kimball et al. 2023). The limited policy interest in informal support interventions may therefore inhibit Government resources for the design, delivery or evaluation of such programmes. Second, charities and NGOs play a key role in designing and delivering DVA interventions but often lack sufficient resources to undertake rigorous evaluation. Third, the evaluation of informal support interventions in workplaces or faith organisations may not be publicly available. Such research may be, for example, only used for internal purposes or potentially deemed sensitive (e.g., concerns about continuing programmes without evidence of impact).
  The EGM identified relatively few interventions targeting the nature and quality of the relationship between informal supporter and victim‐survivor (*n* = 4) (type b) or community‐wide interventions (*n* = 8). As identified above, the evidence is concentrated around interventions that target the victim‐survivor or informal supporters. Set within the socio‐ecological framework of DVA (Heise 1998; World Health Organisations 2021), many ISS interventions included in the EGM are primarily tailored towards individual levels, targeting personal factors that influence how individuals behave in relation to the victim‐survivor or their social networks. For example, training programmes aimed at upskilling informal supporters (type a interventions) are primarily aiming to improve individual knowledge and attitudes (rather than shift the attitudes of a community or challenge societal norms). Therefore, the EGM highlights gaps in evidence pertaining to interventions that are tailored towards specific levels: relationships and community/societal levels, represented by type b and type d interventions, respectively.
  The EGM identified few interventions focused on the nature and quality of the relationship between victim‐survivor and informal supporter (type b). Wider evidence highlights that social responses to disclosures of abuse can be deemed positive or negative (Rivas et al. 2013; Sylaska and Edwards 2014) and so interventions targeting the relationship are critical for improving outcomes for victim‐survivors. Similarly, the EGM points to relatively few interventions tailored towards community or societal levels. Such studies may reflect broader research trends that focus on individuals rather than broader social or cultural forces that shape DVA (DeKeseredy 2021). The socio‐ecological framework highlights that interventions need to target multiple levels to address DVA.
2. *Populations*
  Studies in the EGM primarily reported data for victim‐survivors. As mentioned above, the foregrounding of victim‐survivors in DVA research is important and driven by an ethical imperative to improve outcomes for this group. However, the EGM identified specific population groups that were absent or limited in such samples. The studies in the EGM provide scant data on victim‐survivors in the longer term (leaving relationship for longer than 1 year), ethnic minority groups and male victim‐survivors. Further, the EGM reported limited data for informal supporters alongside victim‐survivors. In particular, research with samples of friends and family members is lacking, which is potentially problematic given the importance attributed to these types of informal supporters by victim‐survivors.
  The samples in the studies represented different parts of the work but North American populations dominated the evidence base (constituting 65% of studies), which is typical of systematic reviews of interventions in the field of DVA (e.g., Trabold 2018). Whilst there were studies, and samples, drawn from the Global South, these populations were only present in a handful of studies.
3. *Study Design *
  The EGM included a diverse set of study designs, with a high proportion of qualitative and mixed method studies. Whilst some of the mixed method approaches may have included experimental designs to assess the effectiveness of interventions, there were relatively few randomised controlled trials (*n* = 11). Whilst there are calls for more RCTs to evaluate the effectiveness of DVA interventions (Feder et al. 2011), there are also concerns that this study design is not optimal or feasible in the field of DVA (Goodman et al. 2017). This debate is relevant to ISS because, as an emerging area of intervention and research, an experimental evidence base may be needed to build understanding and attract funding from policy actors/decision‐makers for intervention development in this area. Yet, ISS interventions are often implemented and evaluated by charities and NGOs who lack the resources to undertake rigorous experimental evaluations and so produce such an evidence base. Moreover, practitioners and wider stakeholders may not view RCTs to be appropriate or feasible, and/or judge the credibility of research on the basis of relevance over methodological rigour (Casey et al. 2020).
4. *Outcomes*
  A high proportion of the data reported on victim‐survivor samples, with an emphasis on their mental health and wellbeing, and help‐seeking behaviours. Evaluating interventions in terms of the emotional health and wellbeing of the victim‐survivor aligns with outcome priorities identified by the wider service user and providers (Powell et al. 2022). Other priority outcomes for DVA such as the emotional health and wellbeing of children or feelings of safety, however, were not captured by the EGM.
  There were major evidence gaps in terms of the outcomes and data for informal supporters. Few studies provided data on this group and the lack of reporting on behaviour/actions, specifically whether they provided support to victim‐survivors during or following an intervention, potentially hinders understanding of effectiveness. The EGM also highlights an evidence gap pertaining to community‐level outcomes.

### 6.3 Potential Biases in the Mapping Process

The search for studies was comprehensive and included a diverse range of sources. However, the search methods did not seek to address the geographical bias in academic publishing and so likely replicated the bias towards evidence from the Global North (Bol et al. 2023). The use of major and specialist databases located in the Global North, together with an English language restriction, limited the opportunities to identify potentially eligible studies from the Global South. Moreover, the search strategy specifically targeted UK‐based sources (such as the database Social Policy and Practice, and websites of DVA organisations in the United Kingdom) and so there may also have been bias towards grey literature published in the United Kingdom. Recommendation: Expand grey literature sources beyond the United Kingdom and incorporate databases and/or relevant journals from the Global South.

The search may have led to an incomplete set of included studies and so present a partial account of interventions. The range of informal support interventions was not known at the start of the EGM and so the search for studies was intentionally broad. The search strings aimed to capture a diversity of interventions across different informal settings. This meant that the terms and keywords used for each concept (informal support AND domestic abuse) were broad rather than specific. ‘Informal support’, for example, was included in the search string but ‘workplace support’ was not. Therefore, the search may not have identified all studies of informal support interventions, particularly those that used only specific or tailored terminology. Given the rapid imperative of the EGM, it was not possible to undertake further, targeted searching (to ensure coverage of key settings such as workplaces and faith organisations) but this would be recommended for future searching for informal support interventions.

Whilst double screening and moderation activities were integrated into the screening processes for a proportion of the work, the limited resources and rapid imperative meant that a single reviewer (KSB.) completed a large proportion of the screening (on title and abstract; and full text) and so her understanding of the eligibility criteria/the review may have unduly shaped the EGM. This may have resulted in ‘coder drift’ where decisions are made that may differ from the wider review team (Polanin et al. 2019) such as the exclusion of studies that other reviewers may have included at either screening stage. Moreover, it may have implicitly skewed the EGM towards informal support interventions as understood by a single reviewer. Recommendation: ensure that the review has sufficient resources and time for screening to be undertaken by multiple members of the team, in parallel and with frequent discussions (see Polanin et al. 2019).

Data may not have been consistently coded or primary data may have been missed. The coding for each included study for the EGM was undertaken independently by two reviewers, who then agreed to the final version of the coding. However, there may have been two potential chAllenges with the coding process. First, not all outcomes/primary data may have been captured by the coding. The inclusion of qualitative studies and data meant that the coding process for in‐depth, rich studies was difficult to complete in a timely fashion. Whilst all attempts were made to identify all relevant outcome types, the nuances of some data may have been overlooked and/or mischaracterised. Second, some aspects of the coding tool were challenging to apply (such as ‘type of intervention’), so coding decisions may not have been applied consistently. Recommendation: more specific and detailed instruction for each code a priori using lessons learned may help to strengthen the consistency of coding decisions. Allocating more coding time for qualitative studies to ensure that all data is identified and coded appropriately.

### 6.4 Limitations of the EGM

To our knowledge, this EGM is the first to provide a comprehensive and rigorous overview of the evidence on informal support interventions in domestic abuse. Three key limitations are worthy of note.

First, the EGM did not undertake a quality appraisal of primary studies, so it is not possible to draw inferences about the rigour of included studies and whether they can offer insight into the effectiveness or understanding of interventions.

Second, the pre‐specified framework on which the main conclusions are based may potentially oversimplify our understanding of informal support interventions. The framework was helpful to theorise the potential types of intervention and differentiate between them. However, at times, the framework was difficult to apply and/or categorising an intervention according to a single type may have masked the nuance of interventions. Details about the interventions were typically not well reported and some interventions appeared to bridge different types of intervention. Training for Faith leaders, for example, is clearly aimed at informal supporters (type a intervention) but the curriculum could also potentially aim to improve the quality and nature of the relationship with the victim‐survivor (type b intervention). In seeking to categorise interventions as a primary type, the EGM may therefore oversimplify the remit of interventions and the associated evidence. Further, viewing the framework through the lens of the socio‐ecological model highlights that the EGM did not include all potential interventions that were targeted towards societal factors (e.g., social policies that influence the provision of informal support see Vidu et al. 2022). Interventions tailored towards the community (type d) may have addressed structural factors, such as social and cultural gendered norms, but the pre‐specified framework primarily captured interventions that targeted individual, relationship and community‐level factors. Therefore, the socio‐ecological framework (World Health Organisation 2021) may have provided a useful tool for distinguishing between intervention types.

Third, the EGM may have limited applicability to contexts in the Global South. The majority of the evidence, and interventions, included in the EGM originated from the Global North and so were likely shaped by the social, economic, political and cultural contexts of these geographic areas. The high proportion of interventions focused on individuals and their behaviours, for example, may reflect the neoliberal policy context associated with the Global North (Kimball et al. 2023).

### 6.5 Stakeholder Engagement Throughout the EGM Process

An Advisory Group facilitated stakeholder engagement throughout the EGM. See Methods Stakeholder Engagement for the methods and outcomes associated with this engagement.

## 7 Authors' Conclusions

To our knowledge, this EGM is the first to provide a comprehensive and rigorous overview of the evidence on informal support interventions in domestic abuse. The EGM provides a valuable tool for stakeholders seeking to understand more about informal support interventions, whether to inform policy/practice or commission further research.

### 7.1 Implications for Research

The EGM has assembled the extant research on informal support interventions. This provides a first step for identifying priority areas for future research to improve the breadth, depth and rigour of the evidence base. Given the wide remit of the EGM, in terms of intervention type and geography, a relatively small number of primary studies (*n* = 47) were included. There is a scope to develop the evidence base and share learning about informal support interventions by undertaking more primary research in this area. There are also specific pockets of evidence that could be strengthened:

- *Population*: A high proportion of the studies included in the EGM provided empirical data for victim‐survivors, but greater research is required to understand the interaction of informal support interventions with specific groups of victim‐survivors: women who had left the abusive relationship over 1 year ago, male victim‐survivors and victim‐survivors from ethnic minority groups. Moreover, it would be useful to develop further research with samples of informal supporters, including those who identify specifically as friends and family.
- *Outcome*: The EGM included studies that reported on a wide range of outcomes for victim‐survivors of DVA. Future research could helpfully report on outcomes prioritised by service users/providers such as victim‐survivors' feelings of safety (Powell et al. 2022). Extending the outcomes for informal supporters would also be useful to develop an understanding of the behavioural effects of interventions for this group.
- *Research design*: The EGM included relatively few studies that aimed to evaluate the impacts or effectiveness of informal support interventions. Whilst there is debate about the most appropriate method for assessing outcomes in DVA (Feder et al. 2011; Goodman et al. 2017), robust evaluation of interventions would serve to strengthen the evidence base.

Additional steps could also be taken to develop the EGM and its utility. This could include (1) quality appraisal of included studies to identify the strengths and weaknesses of the evidence base, (2) targeted search for research from the Global South and broadening the search of DVA organisation websites and (3) synthesis of evidence on particular intervention types or population groups.

### 7.2 Implications for Policy

The EGM provides policymakers, at local, national and international levels, with a valuable tool for navigating the evidence base and asking questions of research surrounding informal support interventions. Whilst the EGM provides a partial picture of such interventions, the existing evidence base could be harnessed to advance understanding and inform decision‐making in several ways:

- The EGM can provide a source of inspiration for policymakers, primarily in the Global North, wanting to consider the potential of informal support interventions as part of their policy response to domestic abuse. As informal support has received relatively limited attention from policymakers, this EGM can provide a useful starting point for identifying relevant stakeholders and commissioning pilot studies.
- Policymakers can develop a holistic understanding of informal support interventions by engaging with different, complementary sections of the existing evidence base. The body of qualitative research offers insight into the experiences and perceptions of interventions from the perspective of victim‐survivors (*n* = 11) and informal supporters (*n* = 9). Evidence of the effectiveness of interventions is offered by impact evaluations, such as Randomised Controlled Trials (*n* = 11), and a single study provides data on cost‐effectiveness. Engaging with studies from across the evidence base, including mixed method approaches (*n* = 12) can build knowledge about the potential of informal support intervention as part of a diverse policy response to domestic abuse.
- Policymakers can commission further primary and secondary research to strengthen the evidence base on which decisions can be made. Currently, limited resources for research in the field of informal support hinder our understanding of the potential of such interventions. The EGM identifies concentrations of studies that may be amendable to mixed method evidence synthesis such as interventions targeting the community (*n* = 8) or support groups for victim‐survivors (*n* = 19). This would complement existing syntheses on training tailored towards informal supporters (Schucan Bird et al. 2023a, 2024). Investment in a programme of evaluation for informal support interventions would also be useful to build the primary evidence base and address major research gaps. This could include research‐practitioner partnerships with a focus on improving the extent and rigour of the evaluations. In combination, such efforts would provide a basis for enhancing knowledge about informal support in the policy domain.

### 7.3 Implications for Practice

The EGM provides a valuable resource for a wide range of practitioners in specialist and frontline services as well as those working in diverse community contexts. The evidence base offers insight into informal support interventions in different settings, with data available to inform current and future practice:

- Concentrations of evidence provide a source of information for practitioners seeking to commission services and/or interventions. A few examples include: (1) the data on victim‐survivor mental health and wellbeing outcomes provides a useful source of evidence about informal support interventions for public health professionals and those working in specialist support services; (2) evidence from workplaces (*n* = 5) and faith‐based organisations (*n* = 5) offer a valuable source of information on the design and delivery of informal support interventions in such contexts (as well as potential evidence of impact or perceptions of impact). This may be helpful for practitioners seeking to respond to domestic abuse within such sectors; and (3) studies of interventions tailored towards specific population groups, such as ethnic minority women, provide a useful source of data about informal support for victim‐survivors. This can inform the commissioning of interventions and delivery of care by specialist services.
- This EGM includes studies with data from practitioner populations (*n* = 6). Such research can provide insight into the intersection of informal and formal service provision, illustrate potential roles that frontline practitioners can play in facilitating informal support, and provide evidence on effects, or perceptions of effects, of informal support interventions.
- NGOs play a crucial role in developing and delivering innovative informal support interventions. This EGM identified a small evidence base for interventions led by or shaped by such organisations. Yet, many more such interventions have not (yet) been subject to rigorous evaluation and/or such research is not published. To develop learning and share lessons across and beyond the domestic abuse sector, it is imperative to evaluate informal support interventions.

## Author Contributions

- Content: Nicola Stokes, Carol Rivas, Karen Schucan Bird. All authors have expertise in the field of DVA and shaped the substantive focus/analysis of the EGM.
- EGM methods: Karen Schucan Bird, Carol Rivas. KSB and CR led on the development and application of the EGM methods.
- Statistical analysis: Karen Schucan Bird, Carol Rivas. Limited statistical analysis techniques were required for this EGM.
- Information retrieval: Karen Schucan Bird, Carol Rivas, Nicola Stokes. KSB led the development and design of the search strategy. Nicola undertook the majority of the grey literature search. All authors undertook screening of reports.

KSB drafted the report and developed the interactive EGM. All authors read and reviewed the final report.

## Conflicts of Interest

Nicola Stokes was employed at SafeLives, a UK‐wide charity dedicated to ending domestic abuse, at the time of the research project. The other authors declare no conflicts of interest.

## Plans for Updating the EGM

Karen Schucan Bird will be responsible for updating the EGM, aiming to complete an update in the next 5 years.

## Sources of Support

### Internal Sources

- None

No internal sources of support.

### External Sources

- Economic and Social Research Council, UK

This research is funded by the Economic & Social Research Council (ESRC), as part of UK Research & Innovation's rapid response to Covid‐19.

## Differences Between Protocol and Full Report

**Title:** The title has been revised.

**Authors:** Martha Tomlinson made valuable contributions to the Protocol and the research project but did not contribute to the writing up of this EGM for Campbell Systematic Reviews.

**Background:** A new section was added to describe the existing EGMs and/or relevant systematic reviews.

**Objectives:** The objectives were rephrased to adhere to reporting requirements.

**Methods**: Grey literature search sources were changed:

- World Health Organisation violence against women database was not searched. Instead, the search source was added: UN Women Virtual Knowledge Centre to End Violence against Women and Girls.
- World Health Organisation IRIS database was not searched. Instead, the search source was added: World Health Organisation Publications with Health topic ‘Violence against women’.
- European Commission project databases included only searching on two databases, using the terms ‘domestic violence’ or ‘domestic abuse’ on the Regional Policy Projects Database and Horizon Project results platform.

**Framework**: Additional details were added to clarify the framework and how it was applied (i.e., making explicit links to the ecological framework (Heise 1998; World Health Organisation 2021), additional clarity inserted into the data extraction guidelines).

## Supporting information

Supporting information.

Supporting information.
