# Supplementary material for: Interventions for Improving Informal Social Support for Victim‐Survivors of Domestic Violence and Abuse: An Evidence and Gap Map
Source: Campbell Syst Rev. 2025 Apr 16;21(2):e70026. doi: 10.1002/cl2.70026 (PMC12001825; doi:10.1002/cl2.70026)
Supplement: Supplementary file 1 — Supporting information. [file CL2-21-e70026-s001.docx]

# Supporting material 1 to: Interventions for Improving Informal Social Support for Victim-Survivors of Domestic Violence and Abuse: an evidence and gap map

Schucan Bird KL, Stokes N, Rivas C, Tomlinson M
<https://doi.org/10.1002/14651858.CA000279>

# Search strategies

## Search terms APA PsycINFO via Ovid

| ((support system or social support or community support or social network or community network or family support or family involvement or parental support or community involvement or friend support or workplace support or informal social support or peer support) and (domestic violence or partner violence or battered woman or spouse abuse or spousal abuse or ipv or battered or abuses or abusive or violent or pregnant women or domestic abuse or intimate partner violence or partner abuse)).mp. |
| --- |

## Search terms for Social Policy and Practice via Ovid

| ((support system or social support or community support or social network or community network or family support or family involvement or parental support or community involvement or friend support or workplace support or informal social support or peer support) and (domestic violence or partner violence or battered woman or spouse abuse or spousal abuse or ipv or battered or abuses or abusive or violent or pregnant women or domestic abuse or intimate partner violence or partner abuse)).ab. |
| --- |

## Search terms for ASSIA via Proquest

abstract((support system OR social support OR community support OR social network OR community network OR family support OR family involvement OR parental support OR community involvement OR friend support OR workplace support OR informal social support OR peer support) ) AND abstract((domestic violence OR partner violence OR battered woman OR spouse abuse OR spousal abuse OR intimate partner abuse OR intimate partner violence OR battered OR abuses OR abusive OR violent OR pregnant women OR domestic abuse OR intimate partner violence OR partner abuse))

## Search terms for Pub Med

"domestic violence"[MeSH Terms] OR ("domestic"[All Fields] AND "violence"[All Fields]) OR "domestic violence"[All Fields] OR (("partner"[All Fields] OR "partner s"[All Fields] OR "partnered"[All Fields] OR "partnering"[All Fields] OR "partners"[All Fields]) AND ("violence"[MeSH Terms] OR "violence"[All Fields] OR "violence s"[All Fields] OR "violences"[All Fields])) OR ("battered women"[MeSH Terms] OR ("battered"[All Fields] AND "women"[All Fields]) OR "battered women"[All Fields] OR ("battered"[All Fields] AND "woman"[All Fields]) OR "battered woman"[All Fields]) OR ("spouse abuse"[MeSH Terms] OR ("spouse"[All Fields] AND "abuse"[All Fields]) OR "spouse abuse"[All Fields] OR ("spousal"[All Fields] AND "abuse"[All Fields]) OR "spousal abuse"[All Fields]) OR (("domestic"[All Fields] OR "domestically"[All Fields] OR "domesticate"[All Fields] OR "domesticated"[All Fields] OR "domesticates"[All Fields] OR "domesticating"[All Fields] OR "domestication"[MeSH Terms] OR "domestication"[All Fields] OR "domestications"[All Fields] OR "domestics"[All Fields]) AND ("abusable"[All Fields] OR "abuse s"[All Fields] OR "abused"[All Fields] OR "abuser"[All Fields] OR "abuser s"[All Fields] OR "abusers"[All Fields] OR "abuses"[All Fields] OR "abusing"[All Fields] OR "abusive"[All Fields] OR "abusively"[All Fields] OR "abusiveness"[All Fields] OR "abuse"[All Fields])) OR "ipv"[All Fields] OR ("intimate partner violence"[MeSH Terms] OR ("intimate"[All Fields] AND "partner"[All Fields] AND "violence"[All Fields]) OR "intimate partner violence"[All Fields]) OR ("intimate partner violence"[MeSH Terms] OR ("intimate"[All Fields] AND "partner"[All Fields] AND "violence"[All Fields]) OR "intimate partner violence"[All Fields] OR ("intimate"[All Fields] AND "partner"[All Fields] AND "abuse"[All Fields]) OR "intimate partner abuse"[All Fields]) OR ("spouse abuse"[MeSH Terms] OR ("spouse"[All Fields] AND "abuse"[All Fields]) OR "spouse abuse"[All Fields] OR ("partner"[All Fields] AND "abuse"[All Fields]) OR "partner abuse"[All Fields])

AND

"community networks"[MeSH Terms] OR "psychosocial support systems"[MeSH Terms] OR "social networking"[MeSH Terms] OR "social networking"[MeSH Terms] OR "social support"[Text Word] OR "social network"[Text Word] OR "social system"[Text Word] OR "informal social support"[Text Word]

## Search terms for Social Science Citation Index via Web of Science

TS=(domestic violence or partner violence or spouse abuse or domestic abuse or intimate partner violence or intimate partner abuse or partner abuse)

AND

TS=(support system or social support or community support or social network or community network or family support or family involvement or parental support or community involvement or friend support or workplace support or informal social support or peer support)

## Search terms for systematic review and policy-orientated databases

"domestic abuse" OR "domestic violence" OR "partner violence" OR "partner abuse" OR "spousal abuse" OR "spouse abuse" AND “informal social support” OR “informal support” OR “community support” OR “family support” OR “friends support” OR “workplace support” OR “community network” OR “support system” OR “friends and family” OR “peer support” OR “social network” OR “social networks” OR “social support”

## Search terms for DVA specialist databases and websites

“community network” OR “community support” OR “family support” OR “friends support” OR “friends and family” OR “informal support” OR “peer support” OR “social network” OR “social networks” OR “social support” OR “support system” OR “workplace support”
