# Supplementary material for: Interventions for Improving Informal Social Support for Victim‐Survivors of Domestic Violence and Abuse: An Evidence and Gap Map
Source: Campbell Syst Rev. 2025 Apr 16;21(2):e70026. doi: 10.1002/cl2.70026 (PMC12001825; doi:10.1002/cl2.70026)
Supplement: Supplementary file 2 — Supporting information. [file CL2-21-e70026-s002.docx]

**Mapping**

- 1. Study Population
  *Select all that apply*
  - Victim-Survivors
    *Victim-Survivors of Domestic Violence and Abuse (Any adult or young people who are/ have been experiencing violence and abuse in a current or former intimate relationship).*
    - Exposure to DVA? (at time of study)
      - In abusive relationship
      - No longer in abusive relationship (short term)
        *Up to 1 year*
      - No longer in abusive relationship (Long term)
        *Over 1 year*
      - Not reported
    - What gender?
      - Female
      - Male
      - Non-binary
      - Not reported
    - What ethnicity/ race?
      - Ethnic/ racial minority in the country of study
      - Ethnic/ racial majority in the country of study
      - Not reported
    - What age?
      - Adults
        *18 years old and upwards*
      - Young people and Adolescents
        *Under 18 years*
      - Not reported
    - Migrated population
      *Select if the population includes individuals/ groups who are identified/ recognised as immigrant populations*
  - Informal Supporters
    *Informal social supporters refers to friends, colleagues, neighbours or community members, current non-abusive partners, any family member (including step-family, non-blood relatives, family-in-law) (developed from Gregory et al., 2017 definition).*
    - What type of informal supporter?
      *Sample designed to capture data from these informal supporters*
      - Peer
        *Peer: shared personal experience (Nesta, 2015).*
      - Friend
      - Family member
      - Colleague
      - Faith leader
        *Clergy, religious leaders*
      - Community member
      - neighbour
      - Other (please specify)
      - Not reported
  - Practitioners
    *Practitioners from formal services in state, non-governmental organisations/ third sector and the legal system -police, professional workers, shelters, support workers, and counsellors (Kelly et al, 1996).*
  - Communities
    *Community wide populations (general level population groups that are likely to include range of informal supporters and victim/survivors)*
- 2. Study
  - What was the aim of the study?
    - Explicitly reported (specify)
    - Implicit (specify)
  - What country was study/ intervention set in?
    - UK
    - USA
    - Uganda
    - China
    - Hong Kong
    - Unspecified (online)
    - Canada
    - Portugal
    - Denmark
    - South Africa
    - Australia
    - Sweden
    - Vietnam
    - Mexico
    - Not reported
    - ADD (please specify)
    - Rwanda
    - India
    - Netherlands
    - global
    - Ghana
    - Nepal
  - What continent was the study/ intervention set in?
    - Europe
    - North America
    - South America
    - Africa
    - Asia
    - Australasia
  - What type of data?
    - Qualitative
      *Interviews, focus groups, surveys reporting qualitative data*
    - Quantitative
      *Quasi/Experimental, study reporting quantitative data*
  - What was the study design?
    - 1. Qualitative
    - 2. Quantitative Randomised Control Trial
      *Random assignment of the intervention, including natural experiments.
      MMAT: Randomized controlled clinical trial: A clinical study in which individual participants are allocated to intervention or control groups by randomization (intervention assigned by researchers).*
    - 3. Quantitative non randomised design
      *Studies with comparison group, without random allocation, including regression-based designs (DD, RDD, IV, Matching, cohort, etc.)*
    - 4. Quantitative descriptive
    - 5. Mixed Methods
      *Mixed methods (MM) research involves combining qualitative (QUAL) and quantitative (QUAN) methods. In this tool, to be considered MM, studies have to meet the following criteria (Creswell and Plano Clark, 2017): (a) at least one QUAL method and one QUAN method are combined; (b) each method is used rigorously in accordance to the generally accepted criteria in the area (or tradition) of research invoked; and (c) the combination of the methods is carried out at the minimum through a MM design (defined a priori, or emerging) and the
      integration of the QUAL and QUAN phases, results, and data.*
    - Query
    - Economics/ cost-effectiveness
- 3. Informal Social Support Intervention
  - What is the informal social support intervention?
    - Education/ training
      *Any form of educational or training activities that focused on DVA, with explicit emphasis on responding to victim-survivors (i.e. providing informal support)*
    - Community activities
      *Activities aimed at or seeking to engage the community at large/ the public.*
    - Support group
      *'Support groups are interventions, facilitated by professionals, paraprofessionals or peers (or a combination thereof), designed to provide emotional, psychological, educational and sometimes practical support to groups of individuals who share a problem or situation' (Sullivan, 2012: 3)*
    - Advocacy (with explicit ISS element)
      *An advocate, may be professional, para-professional or volunteer, who explicitly aims to improve social support for victim-survivor.*
    - Policy development
    - Other (specify)
    - Counselling/therapy (with explicit ISS element)
      *Therapy that explicitly aims to strengthen social relationships for the victim-survivor.*
    - Informal adjudication methods
      *This includes restorative justice groups or other methods to adjudicate between victim-survivor and perpetrator.*
    - Mentoring/ befriending
  - What type of intervention? Select one
    - a) Targeting the provider of informal social support
      *ISS interventions that focus on the informal supporters and seek to mobilize existing social support and/ or develop new relationships of support. These include 1) Intervention population: intervention is tailored towards informal supporters with a defined relationship with the victim-survivor (such as friends or colleagues) rather than generic population groups such as 'the community', 2) Intervention target/aim: intervention is targeted towards individual level factors (such as individual knowledge or attitudes) that are associated with enhanced capacity to recognise and respond to victim-survivors.*
    - b) Targeting how the support is provided
      *ISS interventions shape the quality of support and/ or relationship between informal support and DVA victim-survivor. These include 1) Intervention population: These interventions typically target individuals or groups who already have an informal relationship with the victim-survivor, 2) Intervention target/ aim: These interventions are tailored towards the interpersonal relations between the victim-survivor and informal supporter and the nature/ quality of interactions between them (e.g., communications between victim-survivor and members of their informal network).*
    - c) Targeting victim-survivors ability to engage with informal support
      *ISS interventions that focus on victim-survivors’ ability to engage with, and utilise, informal social support. These include 1) Intervention population: DVA victim-survivor, 2) Intervention target/ aim: the intervention aims to maximise victim-survivors' informal networks by focusing on individual, personal level factors of the victim-survivor that shape their informal relationships (such as their emotional capacity to engage with informal networks and/ or their personal situation).*
    - d) Targeting the community in which ISS takes place
      *ISS interventions that target the community within which the informal support takes place. Communities are both physical places/ spaces and forms of social organization within which informal support operates (Mancini et al., 2006) These types of intervention aim to enhance the community response to victim-survivors. These include 1) Intervention population: generic community members, 'community at large', who typically do not have a pre-existing relationship with the victim-survivor, 2) Intervention target/ aim: intervention is targeted towards community (such as social cohesion) and societal factors that facilitate or inhibit the provision of positive informal support (such as norms and values).*
  - Where the informal social support will be/was delivered?
    - Home
    - Online
    - Shelter/ Supported housing
    - Workplace
    - Educational setting
    - Place of Worship
    - Wider community
    - Other (please specify)
    - Not reported
    - Nonprofit organization
  - Who will provide/provided the informal social support?
    - What type of informal supporter?
      *Sample designed to capture data from these informal supporters*
      - Peer
      - Friend
      - Family member
      - Colleague
      - Faith leader
        *Clergy, religious leaders*
      - Other (please specify)
      - Not reported
      - Community member
    - Do they share common language/ culture with victim-survivors?
      - Yes
      - No
      - Not reported
    - Prior experience of DVA?
      - Yes, as victim-survivor
      - Yes, as practitioner/ professional
      - No reported experience
      - Not reported
    - Have they received education or training to provide informal support?
      *This can include education or training delivered as part of the informal support intervention OR prior training/ education provided to the informal supporter.*
      - Yes
      - No
      - Not reported
  - Multi-component intervention
    *The enhancement or provision of informal Social Support is only one aspect of an intervention with multiple elements.*
- 4. Main outcomes/ study tells us about...
  - Victim-Survivors
    - Cognitive (knowledge or attitudes)
      *Knowledge or attitudes about DVA*
      - Awareness, knowledge and understanding of DVA
      - Knowledge of support resources
      - Awareness of healthy relationships
      - Attitudes towards gender roles
    - Behavioural
      *Outcomes relating to victim-survivor's actions to stay in relationship and/ or seek help, obtain support.*
      - Accessing/ obtaining informal social support
      - Informal help seeking
        *e.g. new or extended help seeking from informal sources*
      - Formal help seeking
        *e.g. calls to police*
      - Ongoing involvement with abusive partner
      - Disclosure of DVA
        *e.g. disclosure of DVA to informal or formal supporter (i.e. friend, family, neighbours, religious leader, or professional, practitioner in DVA or health sector)*
      - Desire to make changes in relationship
      - Sexual behaviours
    - Social network
      *Any outcomes/ data reporting on the structure (size, density, composition), function (provision of support/ response) and dynamics (relationship) of the victim-survivor's social support network*
      - Size or strength of informal social network
    - Violence or abuse
      *Experiences or incidences of any form of DVA, reports of abuse or violence.*
    - Characteristics of relationship between victim-survivor and perpetrator
    - Psychosocial outcomes
      *e.g. life satisfaction, social connectedness, self efficacy*
      - Domestic violence self efficacy
        *i.e. self confidence in managing abuse-related difficulties, solving problems, and helping oneself (measure used in Ross, 2013, see p. 38)*
      - Assertiveness
      - Satisfaction with informal social support
    - Mental health outcomes
      *e.g. depression, anxiety, self-esteem*
    - Physical health outcomes
    - Employment or education outcomes
    - Economic outcomes
    - Parenting outcomes
    - Housing outcomes
    - Culturally specific outcomes/ needs
  - Informal Social Supporters
    - Cognitive (knowledge or attitudes)
      - Awareness and Understanding of DVA
      - Knowledge of how to respond and support resources
      - Confidence/Motivation in providing ISS
      - Knowledge about emotional resilience/ supporting own well-being
    - Behavioural
      - Provision of informal support
      - Social reaction to disclosure
    - Psychosocial outcomes
      *e.g. perception of social support, Quality of Life, self-esteem*
    - Other (please specify)
  - Community level
    *Outcomes measured for a population/ community group, rather than measured at individual level, and representing changes/ activities across the community.*
    - Awareness and understanding of DVA
      *Recognition of intimate partner violence and abuse; different types*
    - Social acceptance of DVA
      *Acceptability of DVA in a community or society.*
    - Provision of informal social (community) support/ taking action
    - Confidence in providing ISS
    - Attitudes towards gender roles
    - Intimate relationships (experiences, views)
      - Child witness DVA
  - Cost estimates
